# BdWRKY38 is required for the incompatible interaction of *Brachypodium distachyon* with the necrotrophic fungus *Rhizoctonia solani*

**DOI:** 10.1111/tpj.14976

**Published:** 2020-09-19

**Authors:** Yusuke Kouzai, Minami Shimizu, Komaki Inoue, Yukiko Uehara‐Yamaguchi, Kotaro Takahagi, Risa Nakayama, Takakazu Matsuura, Izumi C. Mori, Takashi Hirayama, Sobhy S. H. Abdelsalam, Yoshiteru Noutoshi, Keiichi Mochida

**Affiliations:** ^1^ Bioproductivity Informatics Research Team RIKEN Center for Sustainable Resource Science 1‐7‐22 Suehiro‐cho Tsurumi, Yokohama 230‐0045 Japan; ^2^ Kihara Institute for Biological Research Yokohama City University 641‐12 Maioka‐cho Totsuka, Yokohama 244‐0813 Japan; ^3^ Graduate School of Nanobioscience Yokohama City University 22‐2 Seto, Kanazawa‐ku Yokohama Kanagawa 236‐0027 Japan; ^4^ Institute of Plant Science and Resources (IPSR) Okayama University 2‐20‐1 Chuo Kurashiki 710‐0046 Japan; ^5^ Graduate School of Environmental and Life Science Okayama University 1‐1‐1 Tsushimanaka Okayama 700‐8530 Japan; ^6^ Microalgae Production Technology Laboratory RIKEN Baton Zone Program RIKEN Cluster for Science, Technology and Innovation Hub 1‐7‐22 Suehiro‐cho, Tsurumi‐ku Yokohama Kanagawa 230‐0045 Japan

**Keywords:** *Brachypodium distachyon*, disease resistance, *Rhizoctonia solani*, salicylic acid, incompatible interaction, sheath blight, transcriptome, WRKY

## Abstract

*Rhizoctonia solani* is a soil‐borne necrotrophic fungus that causes sheath blight in grasses. The basal resistance of compatible interactions between *R. solani* and rice is known to be modulated by some WRKY transcription factors (TFs). However, genes and defense responses involved in incompatible interaction with *R. solani* remain unexplored, because no such interactions are known in any host plants. Recently, we demonstrated that Bd3‐1, an accession of the model grass *Brachypodium distachyon*, is resistant to *R. solani* and, upon inoculation with the fungus, undergoes rapid induction of genes responsive to the phytohormone salicylic acid (SA) that encode the WRKY TFs BdWRKY38 and BdWRKY44. Here, we show that endogenous SA and these WRKY TFs positively regulate this accession‐specific *R. solani* resistance. In contrast to a susceptible accession (Bd21), the infection process in the resistant accessions Bd3‐1 and Tek‐3 was suppressed at early stages before the development of fungal biomass and infection machinery. A comparative transcriptome analysis during pathogen infection revealed that putative WRKY‐dependent defense genes were induced faster in the resistant accessions than in Bd21. A gene regulatory network (GRN) analysis based on the transcriptome dataset demonstrated that *BdWRKY38* was a GRN hub connected to many target genes specifically in resistant accessions, whereas *BdWRKY44* was shared in the GRNs of all three accessions. Moreover, overexpression of *BdWRKY38* increased *R. solani* resistance in Bd21. Our findings demonstrate that these resistant accessions can activate an incompatible host response to *R. solani*, and BdWRKY38 regulates this response by mediating SA signaling.

## Significance Statement

*Rhizoctonia solani* is a plant pathogenic fungus that causes sheath blight in grasses. In accordance with its necrotrophic lifestyle with a broad host range, the incompatible plant–*R. solani* interactions are not known in any host plants. Here, we demonstrate that such interactions could occur in the small grass *Brachypodium distachyon*, and BdWRKY38 is a key transcription factor that positively regulates this interaction by mediating the salicylic acid signaling pathway.

## INTRODUCTION

*Rhizoctonia solani* is a soil‐borne necrotrophic fungus that causes disease in various plant species, including the economically important crops rice (*Oryza sativa*), wheat (*Triticum aestivum*), barley (*Hordeum vulgare*), maize (*Zea mays*), potato (*Solanum tuberosum*) and cotton (*Gossypium hirsutum*; Zheng *et al*., 2013; Okubara *et al*., 2014). In particular, *R. solani*, which belongs to anastomosis group AG‐1 1A, is the causal agent of sheath blight, which can devastate rice production in paddy fields. The primary inoculum of sheath blight is sclerotia, masses of hyphae that can remain dormant in soil for years. During field preparation and water management in paddy fields, the sclerotia float and attach to rice leaf sheaths. At this point, the sclerotia germinate and produce oval gray lesions. Once the pathogen has colonized the entire rice plant, it severely impairs plant growth and reduces yield. Because yield losses due to sheath blight represent an estimated 8–50% of total rice production in Asia annually (Okubara *et al*., 2014), managing this pathogen has been a long‐standing challenge in rice production.

The phytohormone salicylic acid (SA) plays a central role in plant immunity. The SA signaling pathway is involved in various defense responses, such as the production of antimicrobial molecules, defense gene expression and hypersensitive responses during incompatible plant–pathogen interactions (Vlot *et al*., 2009). Recently, we demonstrated that exogenous application of SA confers resistance to *R. solani* in both rice and the small model grass *Brachypodium distachyon* (Kouzai *et al*., 2018). Transgenic rice plants overexpressing the bacterial SA‐degrading enzyme NahG were more susceptible than wild‐type plants to *R. solani*. Therefore, SA‐dependent immunity contributes to basal resistance against *R. solani* in grasses. Because SA plays crucial roles in plant resistance against both biotrophic and hemibiotrophic pathogens, these findings suggest that *R. solani* may have a short biotrophic stage during early infection. This hypothesis is supported by the observation that green‐tissue‐specific heterologous expression of Arabidopsis (*Arabidopsis thaliana*) *NPR1* (*NONEXPRESSOR OF PATHOGENESIS‐RELATED 1*), which encodes a master regulator of the SA signaling pathway, enhances sheath blight resistance in a susceptible rice cultivar without negatively affecting growth and yield parameters (Molla *et al*., 2016).

Members of the plant‐specific WRKY transcription factor (TF) family regulate a variety of biological processes, including immunity (Phukan *et al*., 2016). WRKY TFs are characterized by the highly conserved DNA‐binding WRKY domain, which recognizes the W‐box element (TTGAC/T) in the promoter regions of target genes. To date, many WRKY TFs have been identified and their functions elucidated in various plant species. In Arabidopsis, AtWRKY70, one of the best‐characterized WRKY TFs in plants, contributes to resistance against bacterial and fungal pathogens, including the necrotrophic fungus *Botrytis cinerea* (Li *et al*., 2006; AbuQamar *et al*., 2006).

Among the WRKY TFs present in grass species, those involved in disease resistance have been investigated mainly in rice. OsWRKY13, OsWRKY22, OsWRKY31, OsWRKY45, OsWRKY47, OsWRKY51, OsWRKY53, OsWRKY67 and OsWRKY89 are positive regulators of disease resistance against the causal agents of rice blast (*Magnaporthe oryzae*) and/or bacterial leaf blight (*Xanthomonas oryzae* pv. *oryzae*, *Xoo*; Qiu *et al*., 2007; Shimono *et al*., 2007; Wang *et al*., 2007; Zhang *et al*., 2008; Abbruscato *et al*., 2012; Wei *et al*., 2013; Chujo *et al*., 2014; Hwang *et al*., 2016; Liu *et al*., 2018). Knockout of *OsWRKY22* and RNA interference (RNAi)‐based silencing of *OsWRKY67* compromise resistance against incompatible strains of *M. oryzae* and *Xoo*, suggesting that the major roles of these WRKY TFs are to promote disease resistance (R) protein‐mediated defense responses. *OsWRKY47* is strongly induced during incompatible rice–*M. oryzae* interactions, and its overexpression increases resistance to a compatible strain of *M. oryzae*. By contrast, OsWRKY62 and OsWRKY76 negatively regulate disease resistance (Peng *et al*., 2008; Yokotani *et al*., 2013; Liu *et al*., 2016). Overexpression of *OsWRKY62* or *OsWRKY76* reduces resistance to both compatible and incompatible strains of *M. oryzae* and *Xoo*, even though these genes are upregulated in response to the pathogens. OsWRKY28 was also identified as a negative regulator of resistance to a compatible strain of *M. oryzae* (Chujo *et al*., 2013). Therefore, the WRKY TFs have diversified functions in disease resistance, such that they can positively or negatively regulate defense responses to compatible and incompatible pathogens.

Genes and defense responses involved in incompatible interactions with *R. solani* are largely unknown because no such interactions have been observed in any host plant, including rice (Hashiba, 1984). However, some WRKY TFs have been shown to modulate defense responses during compatible interactions with *R. solani*. In cotton, *GhWRKY27a* and *GhWRKY39‐1* may be related to basal resistance, because their heterologous expression in *Nicotiana benthamiana* alters resistance against compatible strains of *R. solani* (Shi *et al*., 2014; Yan *et al*., 2015). In rice, overexpression of *OsWRKY4*, *OsWRKY13*, *OsWRKY30* and *OsWRKY80* increases the *R. solani* resistance of susceptible cultivars (Peng *et al*., 2012, 2016; Wang *et al*., 2015; John Lilly and Subramanian, 2019). However, we established that two accessions of *B. distachyon*, Bd3‐1 and Gaz‐4, exhibit strong resistance to *R. solani* (i.e. accession‐specific resistance). Bd3‐1 showed upregulation of SA marker genes encoding WRKY TFs within 5 h post‐inoculation (hpi), and Gaz‐4 did so at 24 hpi. By contrast, another *B. distachyon* accession, Bd21, is susceptible to *R. solani* and did not show induction of the SA marker genes up to 48 hpi (Kouzai *et al*., 2018). These findings imply that the accession‐specific *R. solani* resistance in *B. distachyon* populations is governed by R protein(s) that mediate incompatible interactions.

A comparison of the defense responses to *R. solani* among *B. distachyon* accessions would be helpful to clarify the molecular mechanisms underlying incompatible plant–*R. solani* interactions. In this study, we characterized accession‐specific *R. solani* resistance, and established that BdWRKY38 positively regulates this disease resistance by mediating the SA signaling pathway.

## RESULTS

### Endogenous SA and its downstream WRKY TFs contribute to *Rhizoctonia solani* resistance in *Brachipodium distachyon* Bd3‐1

To investigate accession‐specific *R. solani* resistance in *B. distachyon* populations, we assessed the involvement of endogenous SA and SA‐inducible WRKY TFs using transgenic plants. In our previous study, we had demonstrated that, in contrast to the susceptible accession Bd21, *B. distachyon* accession Bd3‐1 exhibits strong resistance to *R. solani* isolate MAFF305230 (Kouzai *et al*., 2018). Moreover, Bd3‐1 upregulates two SA‐inducible WRKY genes, *WRKY45L1* and *WRKY45L2* (Kouzai *et al*., 2016), within 5 hpi, and that this response is not observed in Bd21. In this study, to examine whether endogenous SA is required for this *R. solani* resistance, we produced transgenic Bd3‐1 plants heterologously expressing the bacterial SA‐degrading enzyme NahG (*NahG*‐ox; Figure S1a), following a strategy frequently used to produce SA‐deficient plants in various species (Abreu and Munné‐Bosch, 2009). We found that both endogenous SA levels and *R. solani* resistance were significantly lower in the *NahG*‐ox plants (T_2_ generation) than in wild‐type Bd3‐1 (Figures S2 and 1), indicating that the accession‐specific *R. solani* resistance in Bd3‐1 is SA dependent.

**Figure 1:**
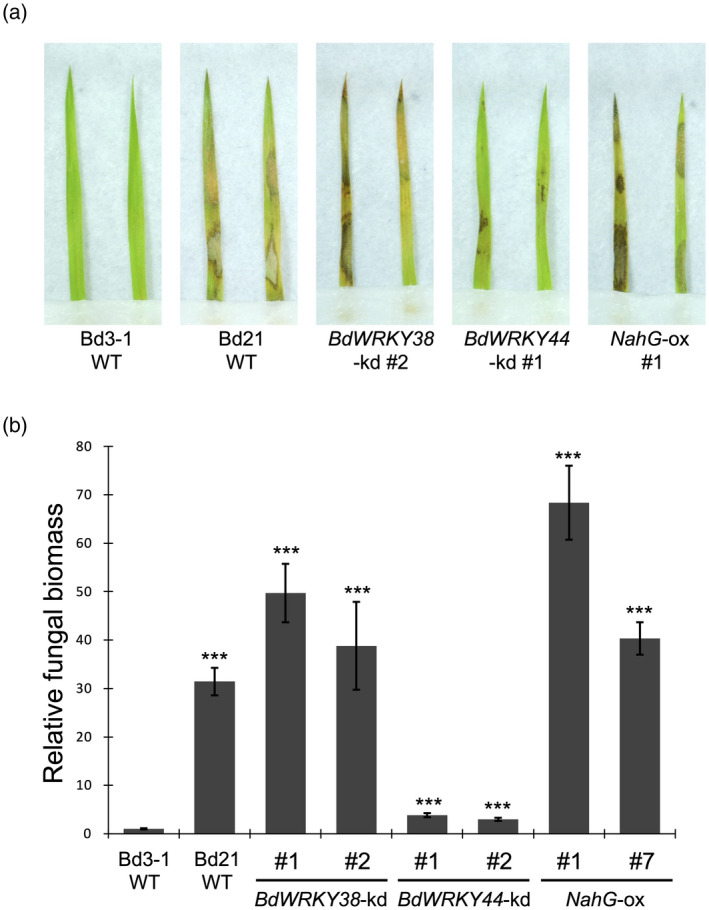
Disease resistance to *Rhizoctonia solani* in *BdWRKY38*‐kd, *BdWRKY44*‐kd and *NahG*‐ox plants. *BdWRKY38*‐kd (knockdown), *BdWRKY44*‐kd and *NahG*‐ox plants [the latter overexpressing the salicylic acid (SA)‐degradation protein NahG] were produced in a *Brachipodium distachyon* Bd3‐1 background. (a) Lesion formation caused by *R. solani* on detached leaves of the three genotypes, along with resistant Bd3‐1 and susceptible Bd21 wild‐type (WT) plants, at 3 days post inoculation (dpi). (b) Relative biomass of *R. solani* in detached leaves at 3 dpi. Data are presented as means ± SEM of values relative to wild‐type Bd3‐1; ***P < 0.001, *n* = 4, using Student’s *t*‐tests. These experiments were performed three times using the transgenic plants at T2 generation, and a representative result is presented.

We next investigated the contribution of two SA‐inducible WRKY genes to this resistance. In the *B. distachyon* Bd21 reference genome, 88 genes encoding putative WRKY TFs are present, according to PlantTFDB. We named these genes based on their chromosome location and position from the 5′ end (Data S1). According to this nomenclature, WRKY45L1 and WRKY45L2, which we had previously designated based on their sequence similarities to rice OsWRKY45 (Kouzai *et al*., 2016), were renamed BdWRKY38 and BdWRKY44, respectively.

To investigate whether BdWRKY38 and BdWRKY44 contribute to the accession‐specific *R. solani* resistance, we analyzed transgenic Bd3‐1 plants, in which the expression of *BdWRKY38* and *BdWRKY44* was suppressed by RNAi‐based gene silencing. We confirmed the reduced expression of both genes in the transgenic plants by quantitative reverse transcription polymerase chain reaction (qRT‐PCR; Figure S1b,c) and obtained two independent knockdown lines (T_2_ generation) for each gene, which we refer to as *BdWRKY38*‐kd and *BdWRKY44*‐kd, respectively. After inoculation with *R. solani*, foliar symptoms and fungal biomass in *BdWRKY38*‐kd plants were greater than those in wild‐type Bd3‐1 (Figure 1), which were comparable to those in the susceptible accession Bd21 at 3 days post‐inoculation (dpi). *BdWRKY44*‐kd plants also exhibited compromised *R. solani* resistance, but the degree of decreased resistance was approximately 10 times lower than that in *BdWRKY38*‐kd plants (Figure 1). Thus, BdWRKY38 plays a major role in the accession‐specific *R. solani* resistance in Bd3‐1.

### *Rhizoctonia solani* infection on *Brachipodium distachyon* Bd3‐1 and Tek‐3 is arrested at an early time point

**Figure 2:**
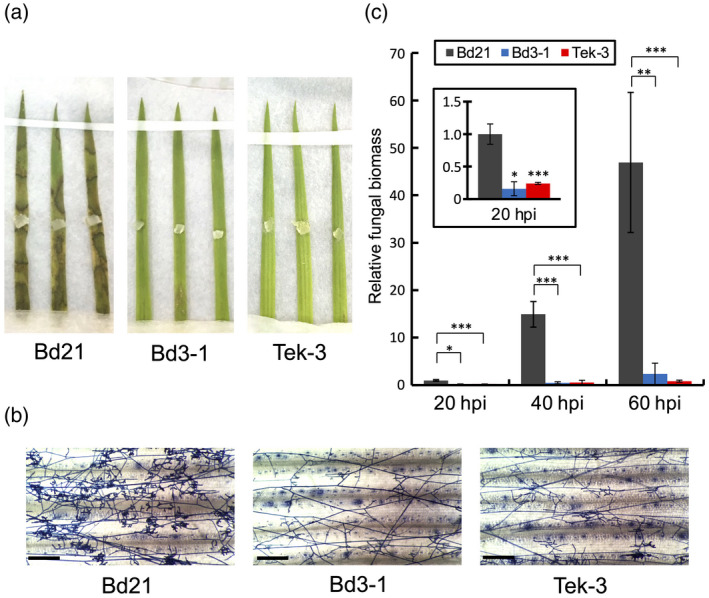
Infection process of *Rhizoctonia solani* in *Brachipodium distachyon* accessions. The *R. solani* infection process was investigated using the *B. distachyon* accessions Bd21, Bd3‐1 and Tek‐3. (a) Lesion formation due to *R. solani* on detached leaves at 3 days post inoculation (dpi). (b) Hyphal growth of *R. solani* on detached leaf surfaces. The inoculated leaves were collected at 1 dpi and the hyphae were stained with trypan blue. Scale bars: 200 μm. (c) Relative biomass of *R. solani* in detached leaves at the indicated time points. Inset shows the data from 20 h post‐inoculation (hpi) on an expanded scale. Data are presented as means ± SEM of values relative to Bd21 at 20 hpi; *P < 0.05, **P < 0.01, ***P < 0.001, *n* = 4, using Student’s *t*‐tests. These experiments were performed three times, and a representative result is presented.

Next, we investigated the infection behavior of *R. solani* on *B. distachyon* accessions through microscopic observation and fungal biomass quantification. In this study, we used Bd3‐1 and a newly identified resistant accession, Tek‐3, because they showed stable and relatively stronger *R. solani* resistance compared with the previously identified resistant accession, Gaz‐4 (Kouzai *et al*., 2018). At 3 dpi, the foliar symptoms were much less pronounced in Bd3‐1 and Tek‐3 than in the susceptible accession Bd21 (Figure 2a). In Bd21 at 1 dpi, *R. solani* had expanded its mycelia throughout the leaf surfaces, and branched hyphae had started to develop specialized infection structures, called infection cushions, that are visually recognizable as aggregates of convoluted hyphae (Figure 2b). By contrast, in Bd3‐1 and Tek‐3 at 1 dpi, the mycelial density was clearly lower than in Bd21, and aggregated hyphae‐forming infection cushions were barely detectable. Foliar fungal biomass in Bd21 increased continuously after inoculation, as demonstrated in our previous report (Kouzai *et al*., 2018), whereas in Bd3‐1 and Tek‐3 it increased very little, remaining significantly lower at all tested time points (Figure 2c). Thus, the progression of *R. solani* infection in Bd3‐1 and Tek‐3 was largely arrested at an early time point.

### *Brachipodium distachyon* accessions recognize *Rhizoctonia solani* within 8 h of infection

We next performed a time‐series transcriptome analysis of Bd21, Bd3‐1 and Tek‐3 during *R. solani* infection to investigate and compare their defense responses. First, we sampled the *R. solani*‐inoculated leaves of each accession at five time points, 0, 4, 8, 16 and 24 hpi, with three biological replicates (Figure S3a). Then, an Illumina‐based RNA sequencing (RNA‐seq) analysis was performed for the 45 samples, and the obtained reads were mapped to the Bd21 reference genome (Table S1).

To explore the datasets, we generated a principal component analysis (PCA) plot and a correlation matrix heatmap. The PCA plot indicated that PC1 clearly separated the time‐series data of each accession, and the data from particular accessions were further separated by PC2 and PC3 (Figure S3b). The correlation matrix heatmap represented the pairwise Pearson correlation coefficients based on the gene expression profiles of each sample, and indicated that transcriptome responses changed substantially between 8 and 16 hpi in all three accessions (Figure S3c). These transcriptome overviews suggest that each accession recognized *R. solani* infection at between 4 and 8 hpi in our experimental conditions.

### Both resistant and susceptible accessions change expression of a similar set of genes during *Rhizoctonia solani* infection

**Figure 3:**
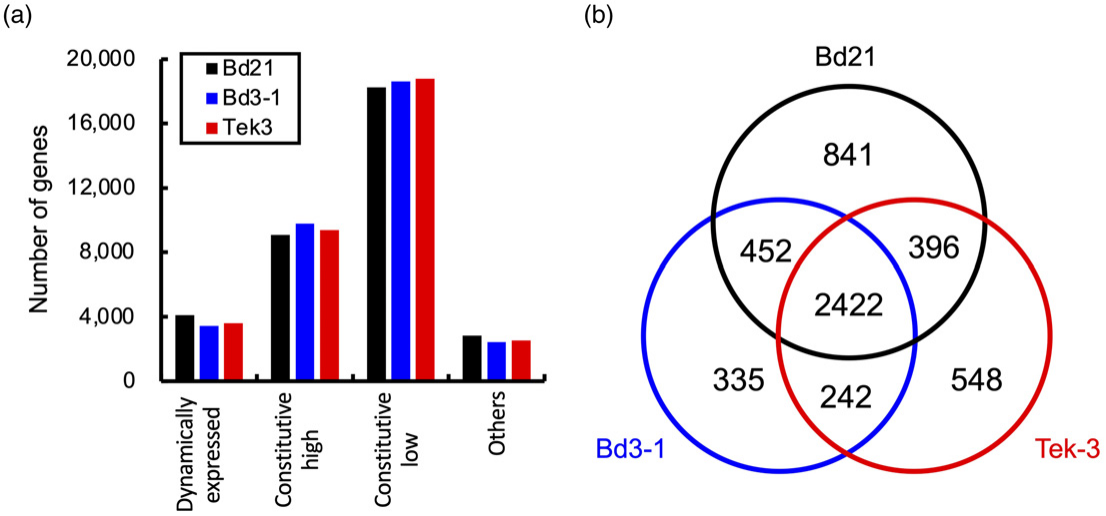
Dynamically expressed genes (DYGs) in *Brachipodium distachyon* after inoculation with *Rhizoctonia solani*. (a) The numbers of DYGs in Bd21 (black), Bd3‐1 (blue) and Tek‐3 (red). DYGs: genes with minimum RPM ≥ 5 and a fold change during the time course (maximum RPM/minimum RPM) ≥ 2. Constitutive high: genes with minimum RPM ≥ 5 and a fold change < 2. Constitutive low: genes with maximum RPM < 5. (b) Proportional Venn diagram showing the overlap of DYGs identified from each accession.

To identify *B. distachyon* genes that are differentially expressed after *R. solani* inoculation in both susceptible and resistant accessions, we examined dynamically expressed genes (DYGs; Levin *et al*., 2016) using our transcriptome datasets. In this study, we defined DYGs as follows: genes with minimum reads per million mapped reads (RPM) ≥ 5, and a fold change during the time course (maximum RPM/ minimum RPM) ≥ 2. We identified 4111, 3451 and 3608 genes as DYGs in Bd21, Bd3‐1 and Tek‐3, respectively (Figure 3a; Data S2–S4). This indicated that approximately 10% of *B. distachyon* genes were significantly responsive to *R. solani* infection in our experimental conditions. We then identified the DYGs that were shared across all three accessions, and determined that 83.2% and 78.1% of the DYGs in Bd3‐1 and Tek‐3 overlapped with those in Bd21 (Figure 3b). Thus, a similar set of genes was responsive to *R. solani* infection in *B. distachyon* accessions regardless of their level of resistance to the pathogen.

### Resistant accessions rapidly induce putative WRKY‐dependent defense genes upon inoculation with *Rhizoctonia solani*

**Figure 4:**
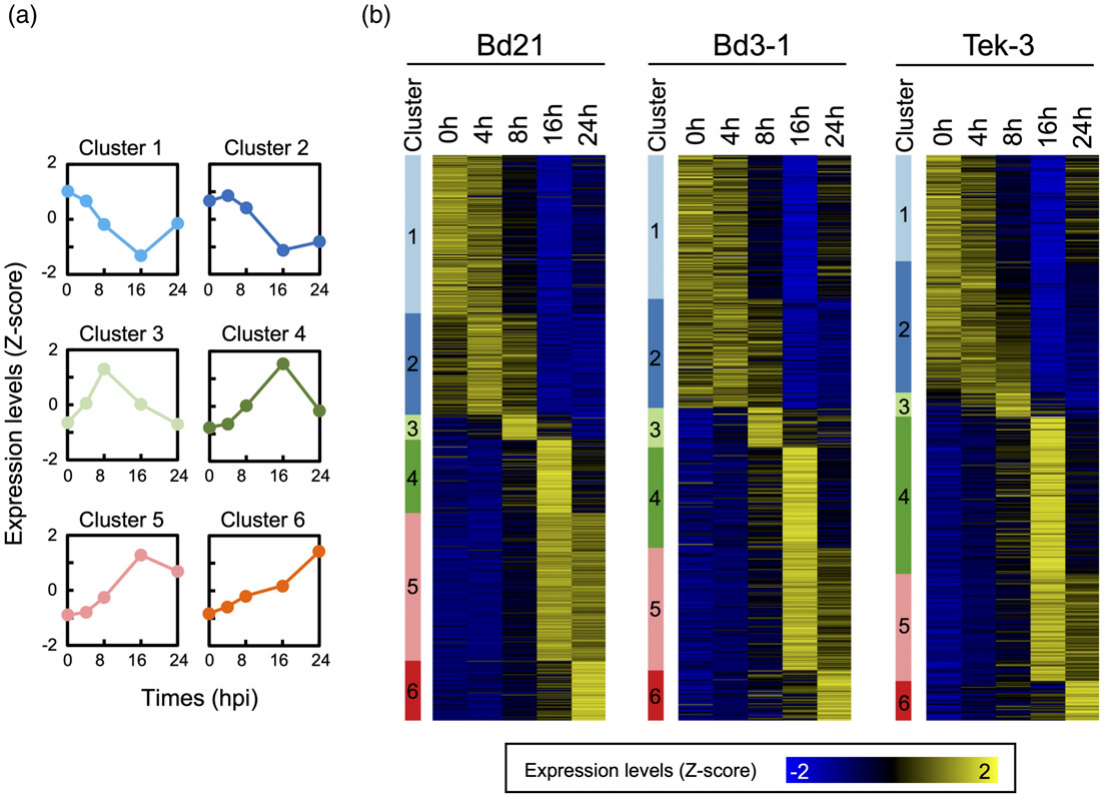
Expression patterns of dynamically expressed genes (DYGs). (a) Time‐series expression levels of DYGs in each cluster. DYGs were subdivided into six clusters based on *k*‐means clustering of their *Z*‐score‐transformed time‐series expression levels in each accession. The expression levels in a particular cluster of each accession were averaged and are presented as line graphs. (b) Expression patterns of DYGs. *Z*‐score‐transformed time‐series expression data of DYGs are visualized by heatmaps in each accession. Yellow indicates positive values; blue indicates negative values; and black indicates zero.

To test the hypothesis that the timing of defense gene expression is associated with *R. solani* resistance in Bd3‐1 and Tek‐3, we analyzed the expression patterns of DYGs across Bd21, Bd3‐1 and Tek‐3. DYGs in each accession were divided into six clusters by *k*‐means clustering based on the time‐series gene expression data (Figure 4a; Data S2–S4). We computed a gap statistic to assess the numbers of clusters and the goodness of clustering, and visualized the DYG expression patterns as heatmaps (Figure 4b). These heatmaps clearly illustrated time‐series transitions in gene expression, whereby all three accessions clearly switched their sets of abundantly expressed genes at 8 hpi. These data were consistent with those in the correlation matrix heatmap (Figure S3c).

**Figure 5:**
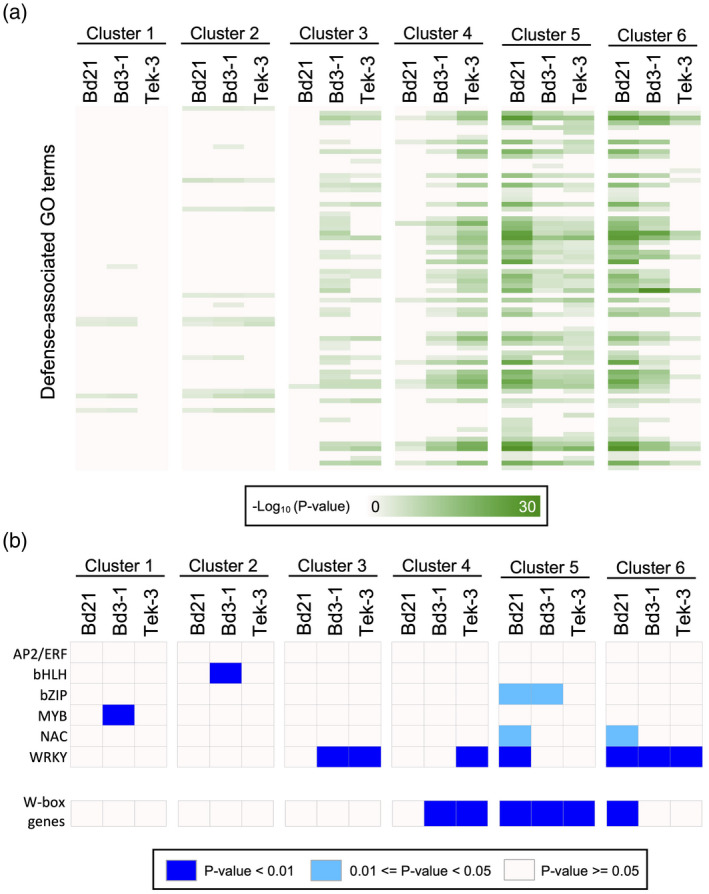
Timing of the expression of genes and transcription factors (TFs) associated with defense responses. (a) Enrichment patterns of gene ontology (GO) terms associated with defense and stress responses. Heatmaps represent the *P*‐values of over‐represented GO terms in each subdivided cluster. The green gradient color scale indicates the −log_10_‐transformed *P*‐value, and white indicates *P*‐values ≥ 0.001. (b) Enrichment patterns of TFs and W‐box elements. Heatmaps represent the *P*‐values of over‐represented TFs and the W‐box elements in each cluster. Blue indicates *P*‐values < 0.01; sky blue indicates *P*‐values ≥ 0.01 and < 0.05; and white indicates *P*‐values ≥ 0.05.

To determine the timing of defense gene upregulation, we performed a gene ontology (GO) enrichment analysis for each cluster of DYGs (Data S5). From the over‐represented GO terms, we selected defense‐associated terms using semantic‐similarity‐based clustering (Figure S4) and examined their patterns of enrichment. In Bd3‐1 and Tek‐3, defense‐associated GO terms were significantly enriched in clusters 3 and 4, which are composed of DYGs abundantly expressed at 8 and 16 hpi. In Bd21, such GO terms were enriched at later stages, including clusters 5 and 6, which are composed of DYGs abundantly expressed at 16 and 24 hpi (Figure 5a).

To explore the TFs that potentially regulate the induction of defense genes after *R. solani* inoculation, we investigated the expression timing of genes encoding particular TFs, which often include regulatory factors involved in plant immunity, such as AP2/ERF, bHLH, bZIP, MYB, NAC and WRKY (Tsuda and Somssich, 2015). We assessed expression timing through an enrichment analysis based on hypergeometric tests, and established that the enrichment patterns of WRKY genes were clearly correlated with the enrichment patterns of defense genes in each accession (Figure 5b). In resistant accessions, WRKY genes were over‐represented among the DYGs from clusters 3, 4 and 6, whereas in Bd21 they were over‐represented among the DYGs from clusters 5 and 6. We also examined the enrichment patterns of genes containing the W‐box (TTGAC/T) in their promoter regions (1 kb upstream of the open reading frame [ORF]), because the W‐box is the cognate cis‐regulatory element for WRKY TFs (Phukan *et al*., 2016). As expected, the enrichment patterns of W‐box genes corresponded to those of defense genes in each accession, and the patterns also differed between resistant and susceptible accessions (Figure 5b). These results strongly suggest that *B. distachyon* defense responses to *R. solani* are primarily regulated by WRKY TFs, and they are activated faster in the resistant accessions Bd3‐1 and Tek‐3 than in the susceptible accession Bd21.

### *BdWRKY38* consists of a hub specifically in the gene regulatory networks (GRNs) of resistant accessions

To further characterize the differences in defense responses to *R. solani* between the resistant and susceptible accessions, we performed a GRN analysis in Bd3‐1, Tek‐3 and Bd21 by setting the DYGs encoding WRKY TFs as potential regulators. GRNs in each accession were inferred from the time‐series gene expression datasets of DYGs using a machine learning‐based algorithm, followed by computations of node degrees and betweenness centralities of WRKY genes (Figures S5–S7). In this study, we defined the WRKY genes constituting hubs in GRNs as follows: betweenness centrality > 0.01 and potential target genes > 5.

**Figure 6:**
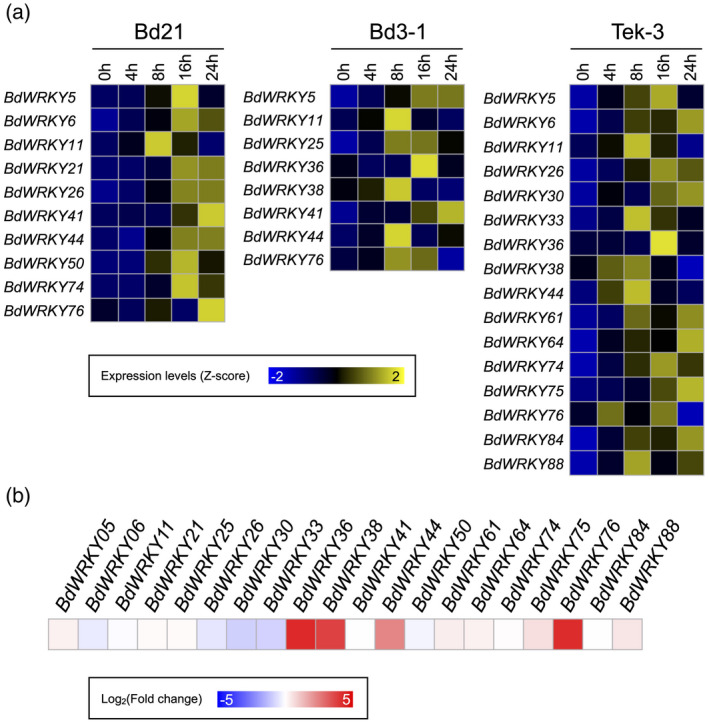
Transcriptional responses of hub WRKY genes to *Rhizoctonia solani* infection and salicylic acid (SA) treatment. (a) The heatmap represents *Z*‐score‐transformed time‐series expression levels of the WRKY genes constituting hubs in the gene regulatory networks (GRNs) of each accession. Yellow indicates positive values, blue indicates negative values, and black indicates zero. (b) SA responsiveness of the hub WRKY genes were visualized by a heatmap. The blue‐red color scale indicates log‐transformed fold changes after 0.5 mm SA treatment in Bd21, based on data retrieved from our previous transcriptome dataset (Kouzai *et al*., 2018).

The GRNs in Bd21, Bd3‐1 and Tek‐3 were composed of 496, 692 and 532 nodes with 10, 8 and 16 hub WRKY genes, respectively. In total, 20 WRKY genes were identified as GRN hubs (Figure 6a; Table S2). Among them, eight WRKY genes were hubs in both susceptible and resistant accessions, and four of these: *BdWRKY5, BdWRKY11*, *BdWRKY44* and *BdWRKY76*, were shared in the GRNs of all three accessions. Two WRKY genes, *BdWRKY21* and *BdWRKY50*, were hubs specifically in the GRN of Bd21. Ten WRKY genes were hubs in the GRNs of the resistant accessions, and two of these, *BdWRKY36* and *BdWRKY38*, were shared between Bd3‐1 and Tek‐3. Among the remainder, *BdWRKY25* was specific to Bd3‐1, and the remaining seven WRKY genes were specific to Tek‐3.

We next investigated the SA responsiveness of these 20 hub WRKY genes using our previously reported transcriptome dataset (Kouzai *et al*., 2018), based on the knowledge that endogenous SA contributes to both basal and accession‐specific resistance to *R. solani* (Figure 1). We established that *BdWRKY36*, *BdWRKY38*, *BdWRKY44* and *BdWRKY76* were SA‐responsive (Figure 6b). In Bd3‐1 and Tek‐3, *BdWRKY38*, *BdWRKY44* and *BdWRKY76* were upregulated within 8 hpi (Figure 6a), whereas *BdWRKY36* was upregulated at 16 hpi. In Bd21, only two WRKY genes, *BdWRKY44* and *BdWRKY76*, were identified as hubs and upregulated at 16 and 24 hpi, respectively (Figure 6a). These results suggest that *BdWRKY38* is a major modulator of the SA signaling pathway required for accession‐specific *R. solani* resistance in *B. distachyon*.

### Overexpression of *BdWRKY38* confers *Rhizoctonia solani* resistance to *Brachipodium distachyon* Bd21

**Figure 7:**
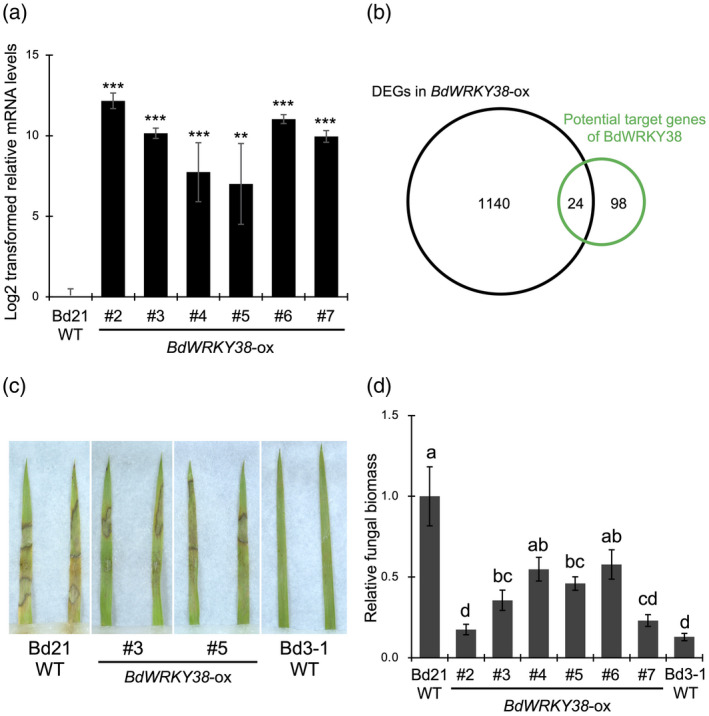
Transcriptional profiling and disease resistance to *Rhizoctonia solani* in *BdWRKY38*‐ox plants. *BdWRKY38*‐ox (*BdWRKY38*‐overexpressing) plants were produced in a *Brachipodium distachyon* Bd21 background. (a) Expression levels of *BdWRKY38* were confirmed by quantitative reverse transcription polymerase chain reaction (qRT‐PCR) in *BdWRKY38*‐ox and wild‐type Bd21. Data are presented as means ± SEM of log_2_‐transformed expression values relative to wild‐type Bd21; **P < 0.01, ***P < 0.001, *n* = 4, using Student’s *t*‐tests. The *BdUbi4* gene was used for normalization. (b) Proportional Venn diagram showing the overlap in the potential target genes of BdWRKY38 inferred from the gene regulatory network (GRN) analysis and the genes differentially expressed in *BdWRKY38*‐ox plants. (c) Lesion formation caused by *R. solani* on detached leaves at 3 days post inoculation (dpi). (d) Relative biomass of *R. solani* in detached leaves at 3 dpi. Data are presented as means ± SEM of values relative to wild‐type Bd21. Different letters above the bars indicate significant differences at *P* < 0.05, *n* = 5, using Tukey’s test. These experiments were performed twice, with similar results using transgenic plants from the T_1_ generation, and representative results are shown.

To further investigate the function of BdWRKY38 in *R. solani* resistance, we generated transgenic plants overexpressing *BdWRKY38* (*BdWRKY38*‐ox) in the Bd21 (susceptible) background and obtained six transgenic lines (T_1_ generation) strongly expressing *BdWRKY38* (Figure 7a). To analyze transcriptome changes in *BdWRKY38*‐ox plants, we performed a 3′ mRNA‐seq‐based transcriptome analysis (Table S3), in which we identified 1164 differentially expressed genes (DEGs) between *BdWRKY38*‐ox and wild‐type Bd21. From the GRNs of Bd3‐1 and Tek‐3 during *R. solani* infection, we identified a total of 180 genes that were potential targets of BdWRKY38. Of these, 118 genes were expressed (read counts > 50) at steady‐state in both *BdWRKY38*‐ox and Bd21. A Venn diagram showed that 24 (20%) of these potential target genes were among the 1164 identified DEGs (Figure 7b; Table S4), suggesting that BdWRKY38 regulates the expression of a portion of the potential target genes in the GRNs at steady‐state. Through the transcriptome analysis, we established that *BdWRKY44* and *BdWRKY76*, the other SA‐inducible hub WRKY genes that were potential targets of BdWRKY38 in the GRNs, were also among DEGs upregulated in *BdWRKY38*‐ox plants (Table S4).

We also evaluated *R. solani* resistance in *BdWRKY38*‐ox plants. At 3 dpi, foliar symptoms on the *BdWRKY38*‐ox plants (T_1_ generation) were relatively weaker than in wild‐type Bd21 but stronger than in wild‐type Bd3‐1 (Figure 7c). We then quantified the fungal biomass in these plants at 3 dpi, and found that it was significantly lower in *BdWRKY38*‐ox plants than in wild‐type Bd21 plants (Figure 7d). Although each of the *BdWRKY38*‐ox lines had a different level of fungal biomass reduction, lines #2 and #7 showed reductions as high as 80% compared with wild‐type Bd21, approaching that of wild‐type Bd3‐1. These results demonstrated that overexpression of BdWRKY38 confers partial *R. solani* resistance to the susceptible *B. distachyon* accession Bd21.

## DISCUSSION

### The incompatible plant–*Rhizoctonia solani* interaction could occur in *Brachipodium distachyon*

The identification of phenotypic variation in sheath blight resistance in *B. distachyon* provides opportunities to elucidate plant defense responses as a mechanism to suppress *R. solani* infection. We previously established that basal resistance to this pathogen depends on endogenous SA, and exogenously applied SA increases resistance in rice and *B. distachyon* (Kouzai *et al*., 2018). In this study, we demonstrated that the accession‐specific *R. solani* resistance found in Bd3‐1 is also SA dependent (Figures 1 and S2). In Bd3‐1, the expression of two SA‐responsive WRKY genes, *BdWRKY38* and *BdWRKY44*, was quickly upregulated after pathogen challenge (Kouzai *et al*., 2018). Thus, the present study clarified that BdWRKY38 has a major role in this disease resistance (Figure 1). In Bd3‐1 and a second resistant accession, Tek‐3, fungal propagation was suppressed within 20 hpi (Figure 2c), and the subsequent development of infection machineries at 1 dpi seen in susceptible strains did not occur (Figure 2b), indicating that the infection process of *R. solani* halts at an early time point in these resistant accessions. Because these responses in Bd3‐1, such as SA‐dependent disease resistance and rapid prevention of pathogen infection, are hallmarks of defense responses mediated by R proteins, these data suggest that the relationship between *R. solani* isolate MAFF305230 and Bd3‐1 (and probably also Tek‐3) qualifies as an incompatible plant–pathogen interaction.

We previously determined that endogenous SA levels in Bd3‐1 do not increase after inoculation with *R. solani* (Kouzai *et al*., 2018). Likewise, in rice and barley inoculated with incompatible pathogens, no SA accumulation occurs in local and systemic tissues, whereas hallmarks of incompatible interaction, such as hypersensitive responses and induction of SA‐responsive genes, are observed (Hückelhoven *et al*., 1999; Jiang *et al*., 2010; Takatsuji, 2014). Moreover, exogenous treatment with SA, or its functional analogs, improves disease resistance to various compatible pathogens, including *R. solani*, in rice, barley and *B. distachyon* (Shimono *et al*., 2007; Walters *et al*., 2014; Kouzai *et al*., 2018; Zhou and Wang, 2018). In dicot plants, activation of the SA signaling pathway during incompatible interactions is directly correlated with cellular accumulation of free SA (Vlot *et al*., 2009; Noutoshi *et al*., 2012). Therefore, activation of SA signaling might not be associated with a dramatic accumulation of endogenous SA in grass plants. The molecular mechanisms underlying this difference between dicot and monocot plants remain elusive.

Our time‐series comparative transcriptome analysis of Bd21, Bd3‐1 and Tek‐3 demonstrated that all three accessions underwent clear changes in their global gene expression patterns after 8 hpi, which included the induction of a similar set of DYGs (Figures 3 and 4). These transcriptome overviews suggest that each accession recognized *R. solani* infection at between 4 and 8 hpi, and may have shifted to a defensive mode. However, functional enrichment analyses highlighted differences in transcription between resistant and susceptible accessions, whereby Bd3‐1 and Tek‐3 showed faster induction of defense genes than Bd21 upon inoculation (Figure 5a). Different expression timing of defense gene activation between susceptible and resistant plants has also been reported in Arabidopsis. For example, in a study of interactions between Arabidopsis and the downy mildew fungus, *Hyaloperonospora arabidopsidis,* Arabidopsis defense‐associated genes were upregulated rapidly (at 1 dpi) after inoculation with an incompatible fungal strain, but more slowly (at 3–5 dpi) after inoculation with a compatible strain (Asai *et al*., 2014). In interactions between Arabidopsis and the bacterial pathogen *Pseudomonas syringae* pv. *tomato*, Mine *et al*. reported that inoculation of an incompatible strain rapidly induces transcriptional reprogramming that included defense‐associated genes, whereas inoculation of a compatible strain delayed the transcriptional response (Mine *et al*., 2018). These findings support the idea that the *R. solani* isolate MAFF305230 is incompatible with the *B. distachyon* accessions Bd3‐1 and Tek‐3.

### BdWRKY38 is a central positive regulator of an incompatible host defense response to *Rhizoctonia solani* in *Brachipodium distachyon*

The present study suggests that WRKY TFs have divergent roles in compatible and incompatible interactions between *R. solani* and *B. distachyon*. The GRN analysis associated with WRKY TFs demonstrated that 20 WRKY genes constitute hubs in the GRNs of Bd21, Bd3‐1 and Tek‐3 (Figure 6a; Table S2). Among them, *BdWRKY21* and *BdWRKY50* were GRN hubs specifically in the susceptible accession, Bd21 (Figure S5), suggesting that they may be involved in the host response to compatible pathogens. *OsWRKY53* and *OsWRKY13* were identified as the closest rice homologs of *BdWRKY21* and *BdWRKY50* (Data S1), respectively. These rice WRKY genes are jasmonic acid (JA)‐responsive and contribute to disease resistance in rice (Chujo *et al*., 2014). *OsWRKY13* also can be induced by SA and ethylene (Qiu *et al*., 2007). We previously reported that Bd21, but not the resistant accessions, specifically activated the JA signaling pathway after *R. solani* inoculation (Kouzai *et al*., 2018). Although the responsiveness of *BdWRKY21* and *BdWRKY50* to phytohormones other than SA remains unclear, they may be involved in JA‐mediated host responses, including wound responses.

The eight WRKY genes identified as common hubs in the GRNs of both resistant and susceptible accessions may be associated with basal resistance to *R. solani* (Figure 6a; Table S2). Among these, *BdWRKY44* and *BdWRKY76* are shared in all three accessions (Table S2; Figures S5–S7), and *BdWRKY44* contributes to *R. solani* resistance (Figure 1). BdWRKY44 is one of the two *B. distachyon* homologs of rice OsWRKY45 (Data S1), a positive master regulator of SA‐dependent disease resistance (Shimono *et al*., 2007). BdWRKY76 is identified phylogenetically close to rice OsWRKY76 (Data S1), a negative regulator of rice defense responses (Yokotani *et al*., 2013; Liu *et al*., 2016). Thus, the intensity and durability of defense responses during basal resistance to *R. solani* may be both positively and negatively regulated by these WRKY TFs. Although the OsWRKY4–OsWRKY80 module regulates rice basal resistance to *R. solani* (Peng *et al*., 2016), the putative counterparts of these two proteins in *B. distachyon*, such as BdWRKY4 and BdWRKY1 (Data S1), were not expressed during *R. solani* infection. This implies that such WRKY module may not be conserved between *B. distachyon* and rice.

The remaining 10 hub WRKY genes specific to the GRNs of the resistant *B. distachyon* accessions may function in the incompatible interactions with *R. solani*. Among them, *BdWRKY36* and *BdWRKY38* were SA‐responsive hubs in both Bd3‐1 and Tek‐3 (Figure 6; Table S2). However, only *BdWRKY38* was highly expressed at 8 hpi during *R. solani* infection (Figure 6a), and *BdWRKY38*‐kd Bd3‐1 plants exhibited dramatically reduced resistance to this pathogen (Figure 1). These results strongly suggest that BdWRKY38 is a central positive regulator of the incompatible plant–*R. solani* interactions in *B. distachyon*. Given that both BdWRKY38 and BdWRKY44 are putative orthologs of OsWRKY45 (Data S1), the *B. distachyon* counterparts of OsWRKY45 may have become sub‐functionalized to play different roles in compatible and incompatible defense responses to *R. solani*.

Overexpression of *BdWRKY38* increased *R. solani* resistance in the susceptible *B. distachyon* accession Bd21 (Figure 7c,d). Contrary to an earlier report that overexpression of *OsWRKY45*, the closest homolog of *BdWRKY38*, did not confer *R. solani* resistance in susceptible rice plants (Shimono *et al*., 2012), our finding suggested that BdWRKY38 positively regulates *R. solani* resistance even in the susceptible *B. distachyon* accessions. This phenotypic difference in the WRKY‐ox plants between rice and *B. distachyon* might be the result of different plant species or target genes due to the sub‐functionalization of duplicated *OsWRKY45* orthologs in *B. distachyon*. Our transcriptome analysis in the *BdWRKY38*‐ox plants revealed that 20% of potential BdWRKY38 target genes, as inferred based on the GRN analysis, were differentially expressed in *BdWRKY38*‐ox plants at steady‐state (Figure 7b), suggesting that *BdWRKY38* may regulate the expression of genes that are responsive to *R. solani* infection. Intriguingly, the expression of *BdWRKY44* and *BdWRKY76*, the other SA‐inducible hub WRKY TFs in *B. distachyon*, was upregulated by factors of 5.1 and 18.4 in *BdWRKY38*‐ox plants, respectively (Table S4). Taking our findings together—the associated upregulation of these three WRKY TFs in response to *R. solani* infection in the resistant accessions (Figure 6a), their SA responsiveness (Figure 6b), and the upregulation of *BdWRKY44* and *BdWRKY76* in the *BdWRKY38*‐ox plants (Table S4)—led us to hypothesize that this trio of WRKY TFs may together act as a regulatory module mediating SA‐dependent defense gene expression in the resistant *B. distachyon* accessions.

Our characterization of accession‐specific *R. solani* resistance in *B. distachyon* illuminated the incompatible plant–*R. solani* interaction. Such interactions may occur in particular plant species even though *R. solani* is a necrotrophic fungal pathogen with a broad host range, and thus no rice cultivars are completely resistant to it (Hashiba, 1984). A similar interaction occurs between tomato (*Solanum lycopersicum*) and its pathogen *Verticillium dahliae*, a soil‐borne necrotrophic fungus that causes vascular wilt disease in over 200 dicot plants. *Verticillium dahliae* shows race‐specific incompatible interactions with specific tomato cultivars that have resistance genes encoding receptor‐like proteins (Kawchuk *et al*., 2001). Moreover, interfamily transfer of the resistance genes conferred *V. dahliae* resistance in Arabidopsis (Fradin *et al*., 2011). Necrotrophic pathogens generally kill host plants and obtain nutrients from dead tissues, whereas biotrophic pathogens deprive nutrients from living host cells (Glazebrook, 2005). Hemibiotrophic pathogens are initially biotrophic and then shift to necrotrophic, with the durations of biotrophy and necrotrophy being dependent on the pathogens. Thus, some necrotrophic pathogens would be expected to employ effector proteins not only to form necrotic lesions but also to suppress host immunity early in infection. Indeed, various small secretory effector‐like proteins are detected in the *R. solani* AG‐1 1A genome (Zheng *et al*., 2013). Genetic exploration of *B. distachyon* populations may make it possible to uncover *R* gene(s) for *R. solani*, which can be utilized as genetic resources to improve sheath blight resistance in grass species.

## EXPERIMENTAL PROCEDURES

### Plant and fungal materials

The *B. distachyon* accessions Bd21, Bd3‐1 and Tek‐3 were originally obtained from the National Plant Germplasm System of the USDA‐ARS. *Brachipodium distachyon* seeds of wild‐type and transgenic plants were germinated on a moist filter paper and 1/2 MS medium containing 1% (w/v) agar and hygromycin B (25 μg ml^−1^), respectively. The germinated seedlings were grown in a growth chamber at 23°C under a 20 h:4 h light/dark photoperiod, as described previously (Kouzai *et al*., 2018). *Rhizoctonia solani* AG‐1, 1A isolate MAFF305230, was obtained from Genebank of the National Agricultural Research Organization (NARO) in Japan and cultivated on potato dextrose agar plates at 23°C, as described previously (Kouzai *et al*., 2018).

### Vector construction and plant transformation

For *BdWRKY38* and *BdWRKY44* knockdown plants, a cDNA fragment located in the 3′ region of each WRKY mRNA was used to trigger RNAi. The fragments were amplified by PCR using the primers listed in Table S5 and cloned into the pENTR/D‐TOPO vector. Then, the fragments were inserted into the pANDA destination vector through an LR clonase reaction with the Gateway cloning system (Miki *et al*., 2005). For the overexpression of *NahG*, its full‐length sequence from *Pseudomonas putida* was retrieved from NCBI and synthesized *de novo* with codon usage optimization. For the overexpression of *BdWRKY38*, its full‐length cDNA sequence (Bradi2g30695) was used. These fragments were amplified by PCR using the primers listed in Table S5, and cloned into the pCAMBIA binary vector between the *B. distachyon* ubiquitin promoter and the nopaline synthase terminator. The vectors for both knockdown and overexpression were transformed into *B. distachyon* Bd3‐1 or Bd21 using the *Agrobacterium tumefaciens*‐mediated method, as described previously (Alves *et al*., 2009).

### Gene expression analysis

Total RNA was extracted from *B. distachyon* leaves using the Nucleospin RNA Plant kit (Takara Bio, Shiga, Japan). cDNA was synthesized using the ReverTra Ace qPCR RT Master Mix with gDNA Remover (TOYOBO, Osaka, Japan). qRT‐PCR was performed using SYBR Premix Ex Taq II (Takara Bio) with the Applied Biosystems 7500 System. A semi‐quantitative RT‐PCR analysis was performed using PrimeSTAR Max DNA Polymerase (Takara). The data were normalized based on the expression of the *BdUbi4* gene (*Bradi3g04730*; Chambers *et al*., 2012). Primers are listed in Table S5.

### Phytohormone measurement

*Brachipodium distachyon* seedlings were cultivated in a growth chamber for 3 weeks. Samples of approximately 400 mg of the aerial parts of the seedlings were used for phytohormone extraction. The content of each phytohormone was quantified by liquid chromatography–tandem mass spectrometry (LC‐MS/MS), as previously reported (Mikami *et al*., 2016).

### Inoculation tests

Detached leaves of *B. distachyon* were used for *R. solani* inoculation tests, as described previously (Kouzai *et al*., 2018). Briefly, the leaves were inoculated with columnar mycelial plugs hollowed out with a biopsy trepan (diameter 3 mm) from the edge of *R. solani* mycelia growing on PDA plates. Fungal propagation in the inoculated leaves was evaluated by quantifying the fungal biomass using qPCR according to previous reports (Sayler and Yang, 2007). *BdFIM* (*Bradi2g13800*) was used for normalization, and the primers used for qPCR are listed in Table S5. The infection behavior of *R. solani* on inoculated leaf surfaces was observed after trypan blue staining using an optical microscope, as previously reported (Kouzai *et al*., 2018).

### Time‐series RNA‐seq analysis

Total RNA was extracted from *R. solani*‐inoculated *B. distachyon* leaves using the Nucleospin RNA Plant kit (Takara Bio) at 0, 4, 8, 16 and 24 hpi with three biological replicates. The quality and quantity of the extracted RNA were evaluated using the NanoDrop OneC (Thermo Fisher Scientific, Waltham, MA, USA) and the 2100 Bioanalyzer (Agilent, Santa Clara, CA, USA). Library preparation for RNA‐seq was performed using the TruSeq RNA library preparation kit (Illumina, San Diego, CA, USA), according to the manufacturer's instructions. Prepared libraries were sequenced using the HiSeq 4000 sequencer (Illumina). The obtained sequence reads were trimmed using Trimmomatic (Bolger *et al*., 2014) and mapped to the *B. distachyon* Bd21 reference genome (Bdistachyon_314_v.3.0) using TopHat v2.1.1 with Bowtie v2.2.6 as its mapping tool (Langmead and Salzberg, 2012). The number of reads mapped to each gene was calculated using FeatureCounts and normalized based on RPM. Genes with minimum RPM ≥ 5 and a fold change during the time course (maximum RPM/minimum RPM) ≥ 2 were defined as DYGs in each accession. *k*‐means clustering of DYGs was performed using the *Z*‐score‐transformed expression datasets with Multiple Experiment Viewer (MeV, 4.9.0) software (Howe *et al*., 2011). The number of clusters and goodness of the clustering were determined based on the gap statistic, which was calculated using the clusGap function of the R package cluster.

### Functional enrichment analysis

Gene ontology enrichment analysis for a set of DYGs was performed as follows. GO terms were assigned to *B. distachyon* genes based on the GO annotations of their closest homologs in Arabidopsis, identified based on a blastp analysis with a threshold *E*‐value < 1e‐5. Significantly over‐represented GO terms categorized to ‘biological process’ with a threshold *P*‐value < 0.001 were identified using the R package GOstats (Falcon and Gentleman, 2007). The over‐represented GO terms were summarized and depicted in a scatter plot based on their semantic similarities using REVIGO (Supek *et al*., 2011). GO terms related to defense and stress response were identified via hierarchical clustering of the summarized GO terms based on their semantic similarities. Enrichment analyses for TFs and the W‐box elements for a set of DYGs were performed based on a hypergeometric test using the R function phyper. The *B. distachyon* genes encoding particular TF families, such as AP2/ERF, bHLH, bZIP, MYB, NAC and WRKY, were retrieved from the PlantTFDB (V5.0) website (Jin *et al*., 2017). The promoter region (1 kb upstream of the ORF) sequences of *B. distachyon* genes were extracted using BEDtools getfasta (version 2.25.0; Quinlan and Hall, 2010). Identification of the W‐box (TTGAC/T) element in each sequence was performed using a custom Perl script.

### GRN analysis

A GRN analysis was performed using the R package GENIE3, which can infer GRNs using a tree‐based machine learning algorithm from gene expression data (Huynh‐Thu *et al*., 2010; Mochida *et al*., 2018). GRNs associated with WRKY genes were constructed using time‐series expression datasets of the DYGs upregulated after *R. solani* inoculation (Clusters 3–6), specifying WRKY genes as candidate GRN regulators. The top‐1000 ranked putative regulatory links were visualized as network graphs using Cytoscape (version 3.7.0), as previously reported (Shannon *et al*., 2003). The centralities of WRKY genes were calculated with Cytoscape, and WRKYs with betweenness centrality > 0.01 and node degree > 5 were defined as hubs in each GRN. The SA responsiveness of *B. distachyon* WRKY genes was retrieved from our previously reported transcriptome dataset (Kouzai *et al*., 2018). Log‐transformed fold changes of RPMs in *B. distachyon* Bd21 after treatment with 0.5 mm SA were presented as a heatmap.

### Phylogenetic analysis

Protein sequences of WRKY TFs in *B. distachyon* were obtained from Phytozome (https://phytozome.jgi.doe.gov/pz/portal.html). The closest homologs of these WRKY TFs in rice and Arabidopsis were identified based on a blastp analysis with a threshold *E*‐value < 1e‐5.

### 3′ mRNA‐seq analysis

Total RNA was extracted from fully expanded leaves of plants overexpressing *BdWRKY38* (*BdWRKY38*‐ox) and wild‐type Bd21 plants using a Nucleospin RNA Plant Kit (Takara Bio) at the 4th‐leaf stage with three biological replicates. Quality and quantity of the extracted RNA were evaluated using the NanoDrop OneC (Thermo Fisher Scientific) and the 2100 Bioanalyzer (Agilent). Library preparation was performed using the QuantSeq 3′ mRNA‐Seq Library Prep Kit (Lexogen, Vienna, Austria), according to the manufacturer's instructions. Prepared libraries were sequenced using the Ion Proton system (Thermo Fisher Scientific). The obtained sequence reads were trimmed using Trimmomatic (Bolger *et al*., 2014) and mapped to the *B. distachyon* Bd21 reference genome (Bdistachyon_314_v.3.0) using BWA MEM as its mapping tool (Li and Durbin, 2009). The number of mapped reads to each gene was calculated using FeatureCounts and normalized based on RPM. A DEG analysis was performed using the R package edgeR (Robinson *et al*., 2010), and the genes with a false discovery rate from Fisher's exact test of < 0.05 and a log_2_‐transformed fold change of > 0.5 or < −0.5 were identified as DEGs.

## DATA ACCESSION NUMBER

The RNA‐seq dataset of the Illumina reads has been submitted to the DNA Data Bank of Japan Sequence Read Archive (https://www.ddbj.nig.ac.jp/dra/index‐e.html) under the accession number DRA008911.

## Author contributions

YK, YN and KM conceived the research and designed the experiments. YK, MS, KI, YU, KT, RN, SA and YN performed the experiments, plant transformation and bioinformatics analyses. TM, IM and TH performed phytohormone measurement. KM supervised the project, and YK, YN and KM wrote the manuscript. All the authors read and approved the final manuscript.

## Conflict of interest

The authors declare no conflict of interest.

## Supporting information


**Figure S1.** Gene expression analysis in transgenic plants.
**Figure S2.** Endogenous levels of phytohormones in *NahG*‐ox plants.
**Figure S3.** Time‐series RNA‐seq analysis of *B. distachyon* accessions after inoculation with *R. solani*.
**Figure S4.** Semantic‐similarity‐based clustering of over‐represented GO terms.
**Figure S5.** GRN graphs inferred in *B. distachyon* Bd21.
**Figure S6.** GRN graphs inferred in *B. distachyon* Bd3‐1.
**Figure S7.** GRN graphs inferred in *B. distachyon* Tek‐3.

**Table S1.** Mapping results of time‐series RNA‐seq reads.
**Table S2.** Hub WRKY genes in GRNs.
**Table S3.** Mapping results of 3' mRNA‐seq reads.
**Table S4.** Overlapped genes between potential target genes of BdWRKY38 during *R. solani* infection and DEGs in BdWRKY38‐ox plants.
**Table S5.** Primers used in this study.

**Data S1.** WRKY TFs in the *B. distachyon* Bd21 genome.
**Data S2.** DYGs in Bd21 after inoculation with *R. solani*.
**Data S3.** DYGs in Bd3‐1 after inoculation with *R. solani*.
**Data S4.** DYGs in Tek‐3 after inoculation with *R. solani*.
**Data S5.** Over‐represented GO terms in each cluster of DYGs.
