# Supplementary material for: BdWRKY38 is required for the incompatible interaction of Brachypodium distachyon with the necrotrophic fungus Rhizoctonia solani
Source: Plant J. 2020 Sep 19;104(4):995–1008. doi: 10.1111/tpj.14976 (PMC7756360; doi:10.1111/tpj.14976)
Supplement: Supplementary file 1 — Figure S1. Gene expression analysis in transgenic plants. Figure S2. Endogenous levels of phytohormones in NahG‐ox plants. Figure S3. Time‐series RNA‐seq analysis of B. distachyon accessions after inoculation with R. solani. Figure S4. Semantic‐similarity‐based clustering of over‐represented GO terms. Figure S5. GRN graphs inferred in B. distachyon Bd21. Figure S6. GRN graphs inferred in B. distachyon Bd3‐1. Figure S7. GRN graphs inferred in B. distachyon Tek‐3. [file TPJ-104-995-s001.pdf]

Supporting figures

Figure S1 Gene expression analysis in transgenic plants.

Expression levels of (a) *NahG* were confirmed by semi-quantitative RT-PCR based on four independent sibling plants in each line. The number of PCR cycles was optimized at 30, and the *BdUbi4* gene was also analyzed as an internal control. Expression levels of *BdWRKY38* and *BdWRKY44* were confirmed by qRT-PCR in (b) *BdWRKY38*-kd and (c) *BdWRKY44*-kd plants. Data are presented as means ± SEM of expression values relative to wild-type Bd3-1; $*P < 0.05$, $n = 4$, using Student's *t*-tests. The *BdUbi4* gene was used for normalization. These experiments were performed twice, with similar results, using transgenic plants from the T_2 generation, and representative results are shown.

Figure S2 Endogenous levels of phytohormones in *NahG*-ox plants.

The content of endogenous phytohormones was measured by liquid chromatography–tandem mass spectrometry (LC-MS/MS) from the aerial parts of *B. distachyon* seedlings in *NahG*-ox (T_2 generation) and wild-type Bd3-1. Results are means ± SEM of six biological replicates. $*P < 0.05$, $**P < 0.01$, $***P < 0.001$, $n = 6$, using Student's *t*-tests. SA, salicylic acid; ABA, abscisic acid; JA, jasmonic acid; JA-Ile, jasmonic acid-isoleucine; tZ, trans-zeatin; iP, isopentenyladenine; IAA, indole acetic acid.

Figure S3 Time-series RNA-seq analysis of *B. distachyon* accessions after inoculation with *R. solani*.

(a) Experimental design of time-series RNA-seq. (b) Principal component analysis of the obtained RNA-seq dataset based on reads per million mapped reads (RPM) of genes. Different colors and shapes or symbols indicate different accessions and time points, respectively. (c) Correlation matrix heatmap presenting pair-wise Pearson correlation coefficients using the obtained RNA-seq dataset based on RPM values of genes. The color scale indicates the degree of correlation, with blue signifying a high correlation between samples.

Figure S4 Semantic-similarity-based clustering of over-represented gene ontology terms.

Significantly over-represented gene ontology (GO) terms categorized as "biological process" were visualized in a 2D semantic-similarity-based space using REVIGO. These GO terms were separated into six clusters based on their positions in semantic space upon hierarchical clustering. Different colors of dots indicate different clusters. The GO terms in cluster C were identified as related to defense and stress responses.

Figure S5 Gene regulatory network graphs inferred in *B. distachyon* Bd21.
The top-1000 ranked putative regulatory links inferred by GENIE3 were visualized as network graphs using Cytoscape. The GRN of Bd21 contained 496 nodes. Blue circles indicate WRKY TFs, and circle sizes represent node degrees.

Figure S6 Gene regulatory network graphs inferred in *B. distachyon* Bd3-1.
The top-1000 ranked putative regulatory links inferred by GENIE3 were visualized as network graphs using Cytoscape. The GRN of Bd21 contained 692 nodes. Blue circles indicate WRKY TFs, and circle sizes represent node degrees.

Figure S7 Gene regulatory network graphs inferred in *B. distachyon* Tek-3.
The top-1000 ranked putative regulatory links inferred by GENIE3 were visualized as network graphs using Cytoscape. The GRN of Bd21 contained 532 nodes. Blue circles indicate WRKY TFs, and circle sizes represent node degrees.

Figure S1

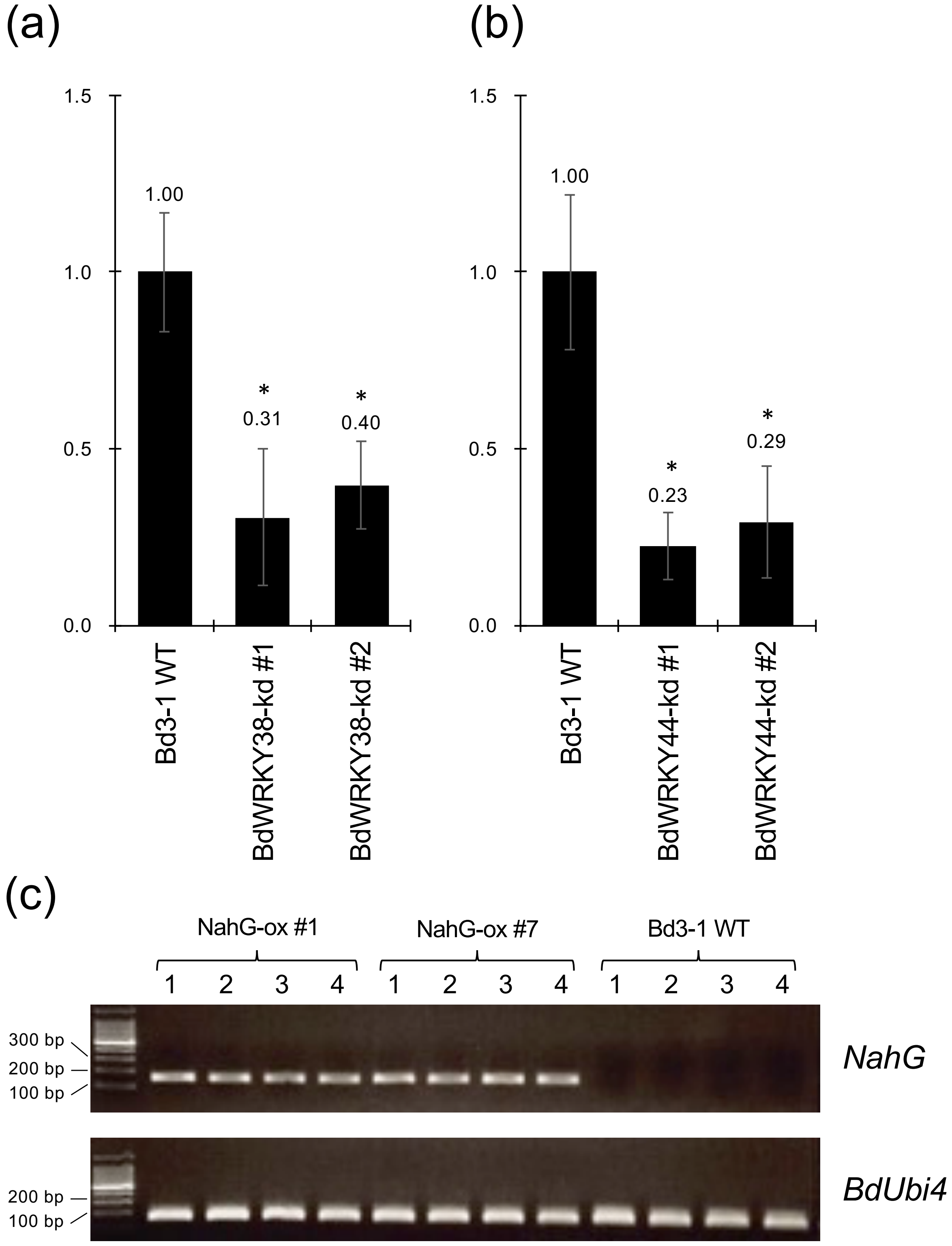

(a)
1.5
1.0
0.5
0.0
1.00
*
0.31
*
0.40
Bd3-1 WT
BdWRKY38-kd #1
BdWRKY38-kd #2
(b)
1.5
1.0
0.5
0.0
1.00
*
0.23
*
0.29
Bd3-1 WT
BdWRKY44-kd #1
BdWRKY44-kd #2
(c)
NahG-ox #1
NahG-ox #7
Bd3-1 WT
1 2 3 4 1 2 3 4 1 2 3 4
300 bp
200 bp
100 bp
NahG
200 bp
100 bp
BdUbi4

Figure S2

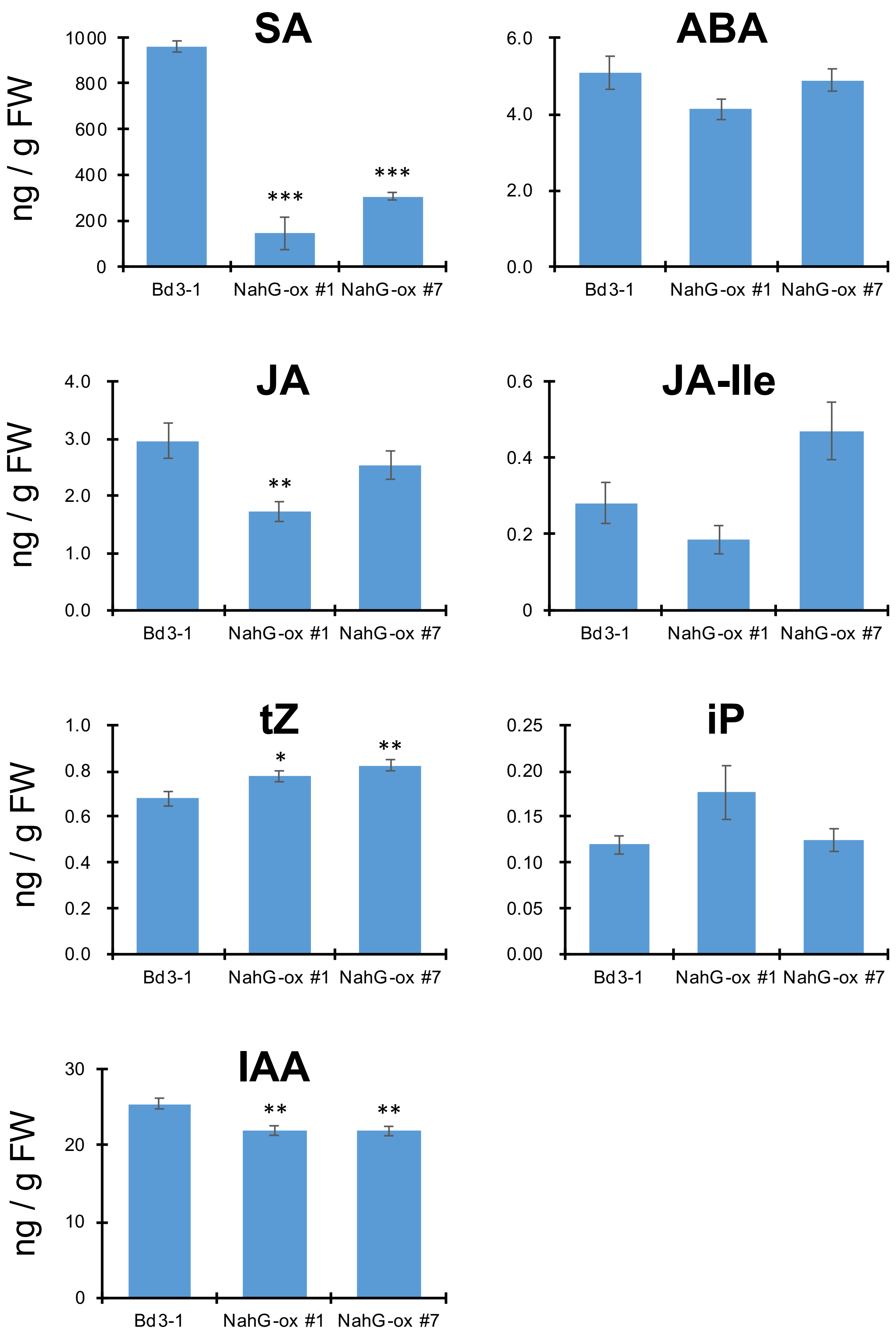

SA
ABA
JA
JA-Ile
tZ
iP
IAA
ng / g FW
1000
800
600
400
200
0
6.0
4.0
2.0
0.0
4.0
3.0
1.0
0.6
0.4
0.2
0.8
0.25
0.20
0.15
0.10
0.05
0.00
30
20
10
***
**
*
Bd3-1
NahG-ox #1
NahG-ox #7

Figure S3

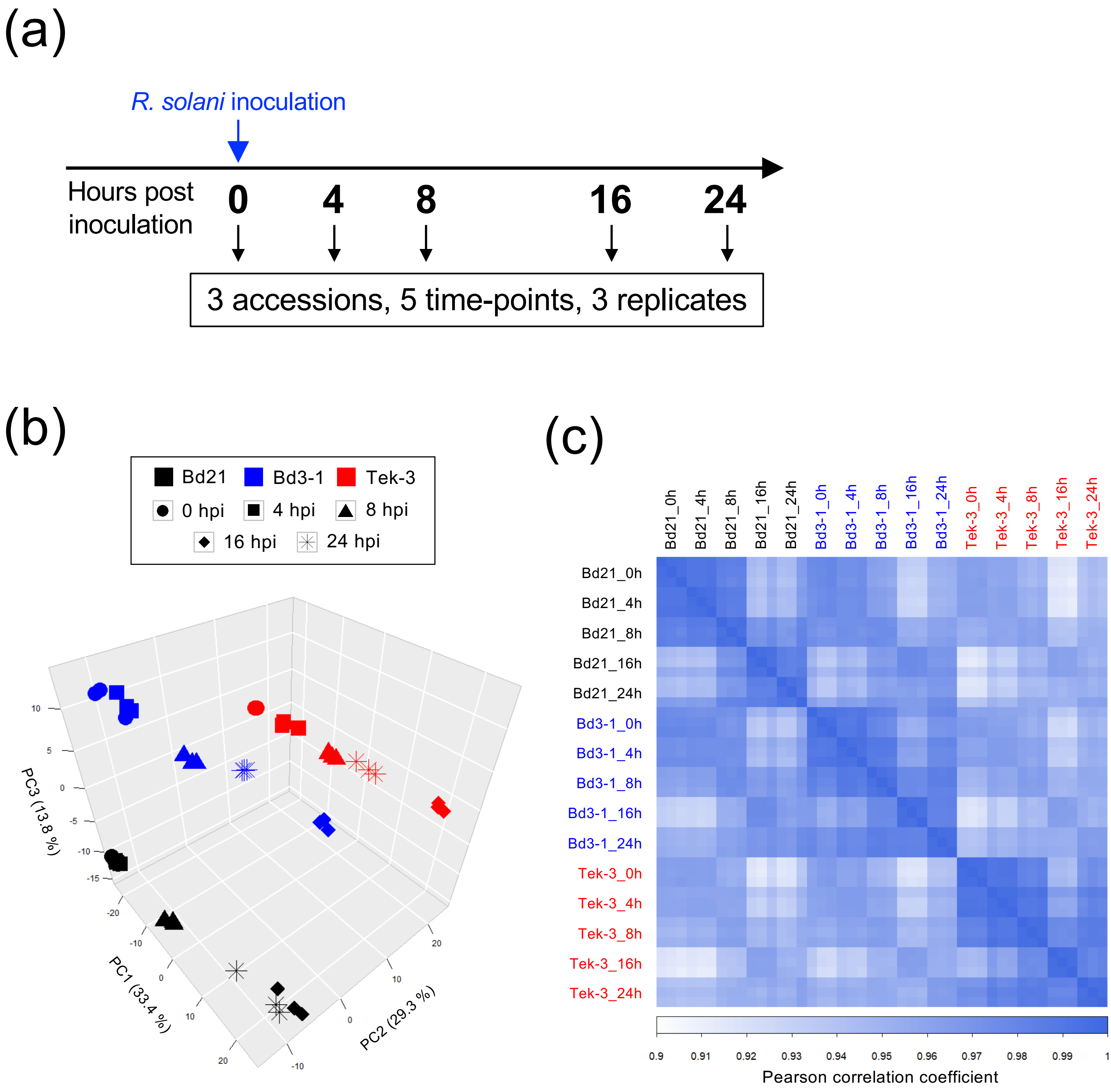
(a)
R. solani inoculation
Hours post inoculation
0
4
8
16
24
3 accessions, 5 time-points, 3 replicates
(b)
Bd21
Bd3-1
Tek-3
0 hpi
4 hpi
8 hpi
16 hpi
24 hpi
PC3 (13.8 %)
PC1 (33.4 %)
PC2 (29.3 %)
(c)
Bd21_0h
Bd21_4h
Bd21_8h
Bd21_16h
Bd21_24h
Bd3-1_0h
Bd3-1_4h
Bd3-1_8h
Bd3-1_16h
Bd3-1_24h
Tek-3_0h
Tek-3_4h
Tek-3_8h
Tek-3_16h
Tek-3_24h
0.9
0.91
0.92
0.93
0.94
0.95
0.96
0.97
0.98
0.99
1
Pearson correlation coefficient

Figure S4

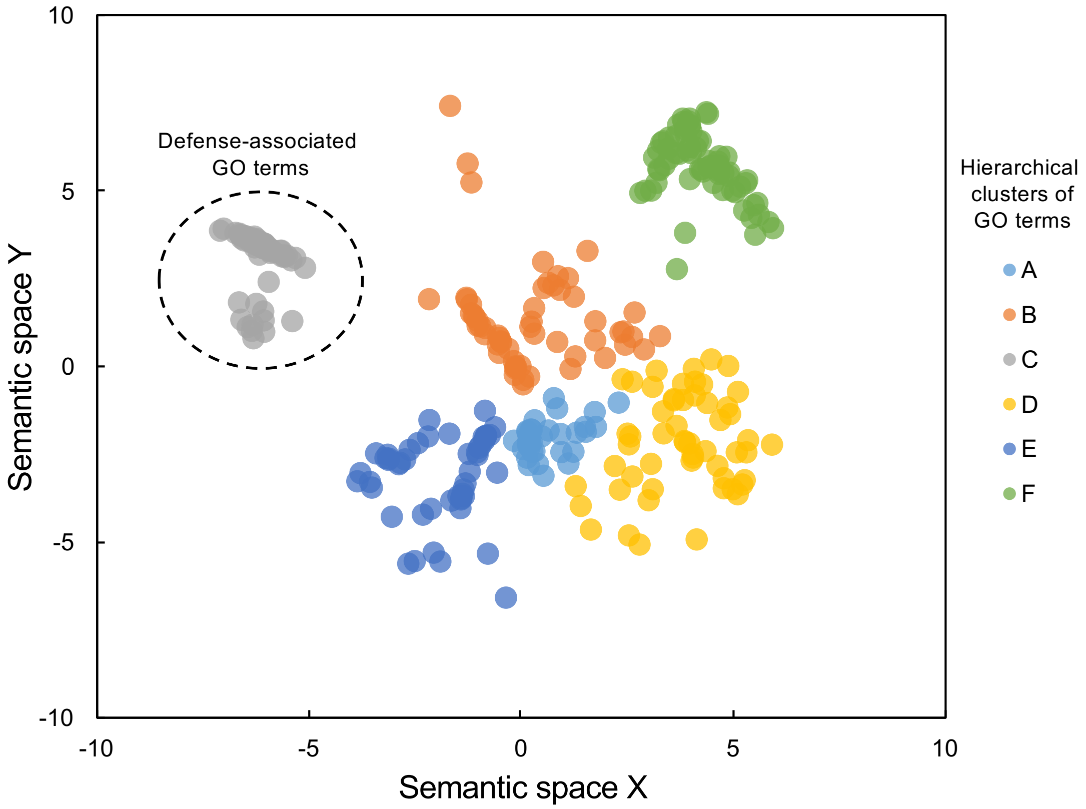

Defense-associated
GO terms
Semantic space Y
Semantic space X
10
5
0
-5
-10
-10
-5
0
5
10
Hierarchical
clusters of
GO terms
A
B
C
D
E
F

Figure S5

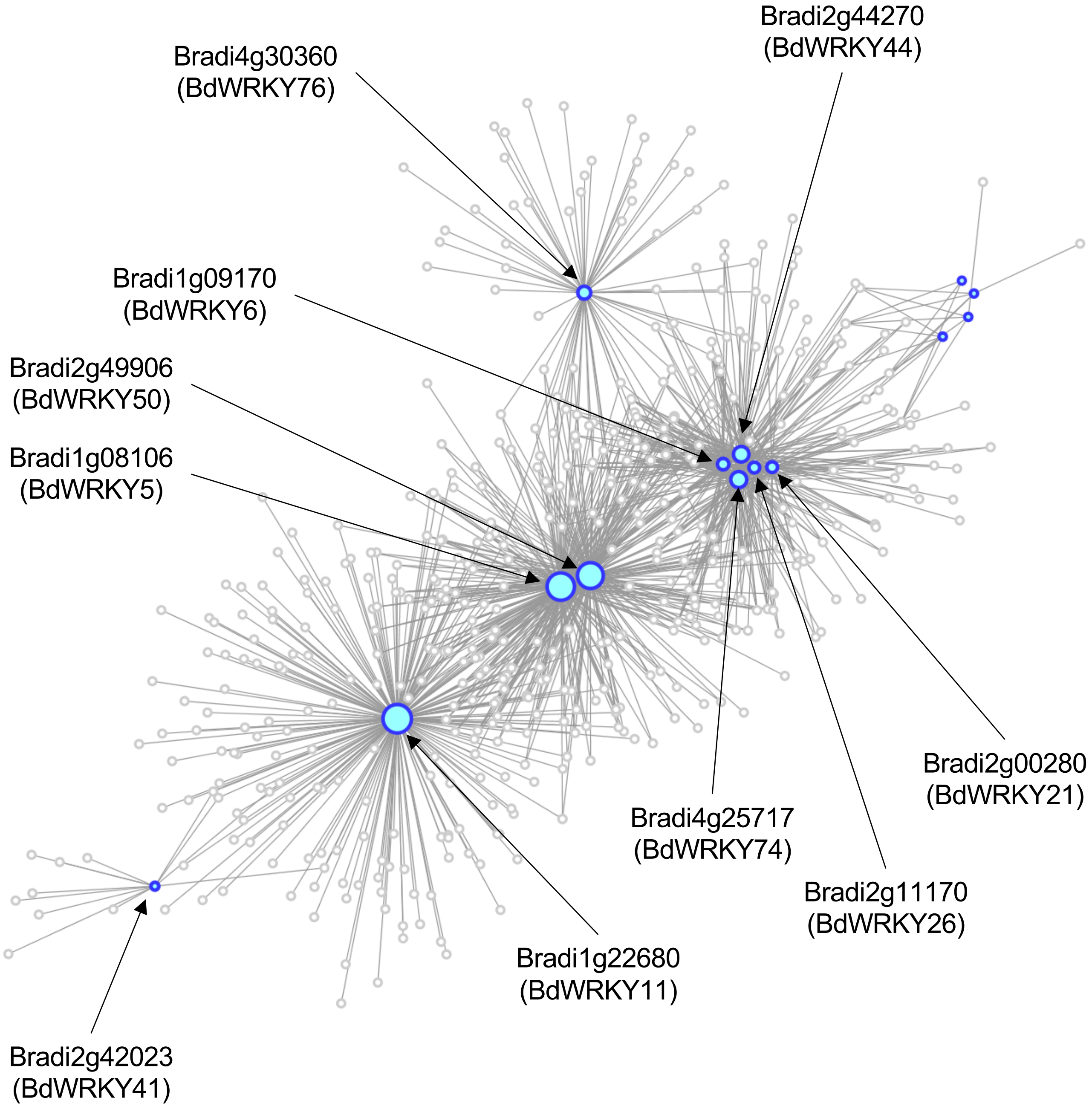
Bradi4g30360
(BdWRKY76)
Bradi2g44270
(BdWRKY44)
Bradi1g09170
(BdWRKY6)
Bradi2g49906
(BdWRKY50)
Bradi1g08106
(BdWRKY5)
Bradi2g00280
(BdWRKY21)
Bradi4g25717
(BdWRKY74)
Bradi2g11170
(BdWRKY26)
Bradi1g22680
(BdWRKY11)
Bradi2g42023
(BdWRKY41)

Figure S6

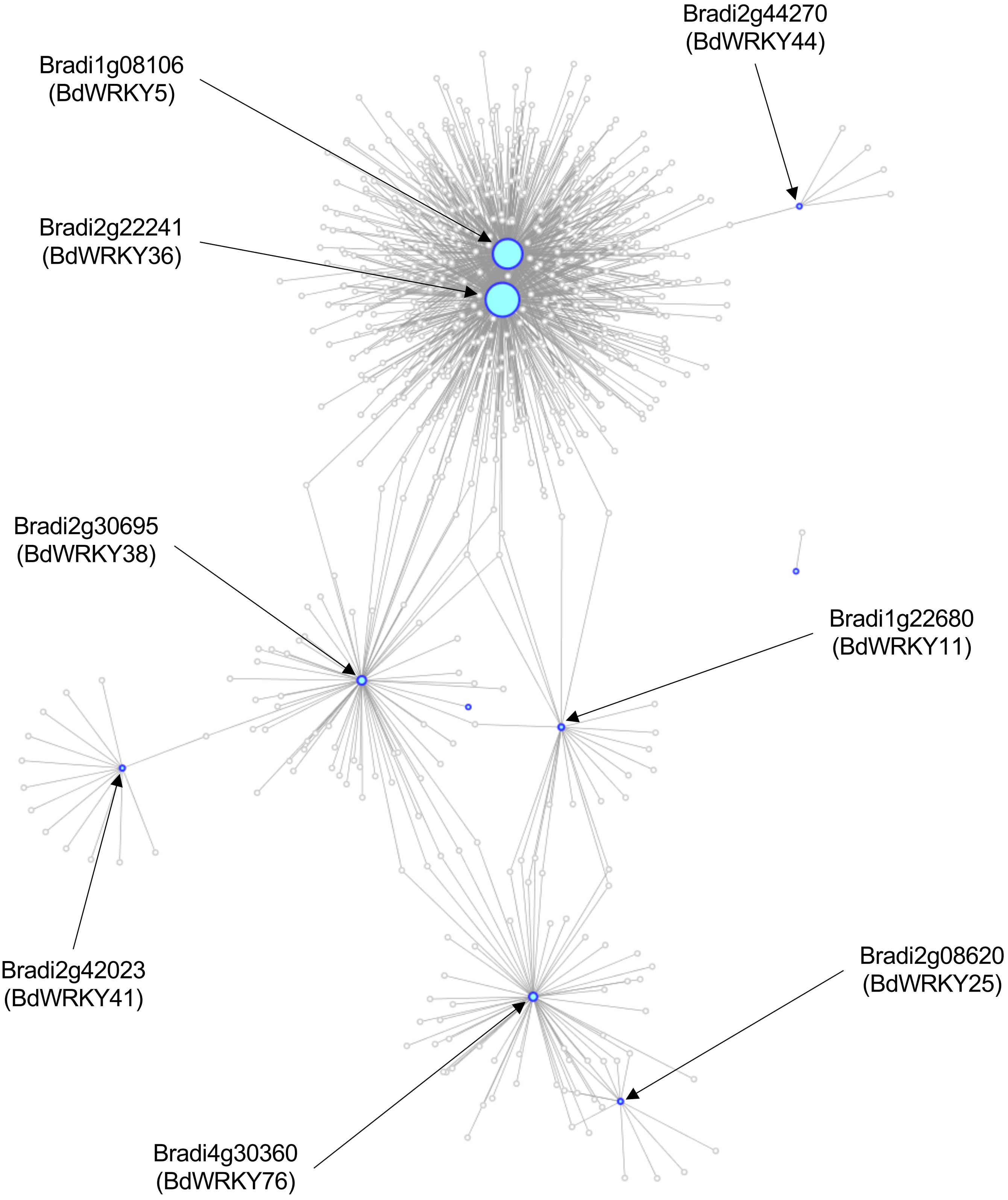

Bradi2g44270
(BdWRKY44)
Bradi1g08106
(BdWRKY5)
Bradi2g22241
(BdWRKY36)
Bradi2g30695
(BdWRKY38)
Bradi1g22680
(BdWRKY11)
Bradi2g42023
(BdWRKY41)
Bradi2g08620
(BdWRKY25)
Bradi4g30360
(BdWRKY76)

Figure S7

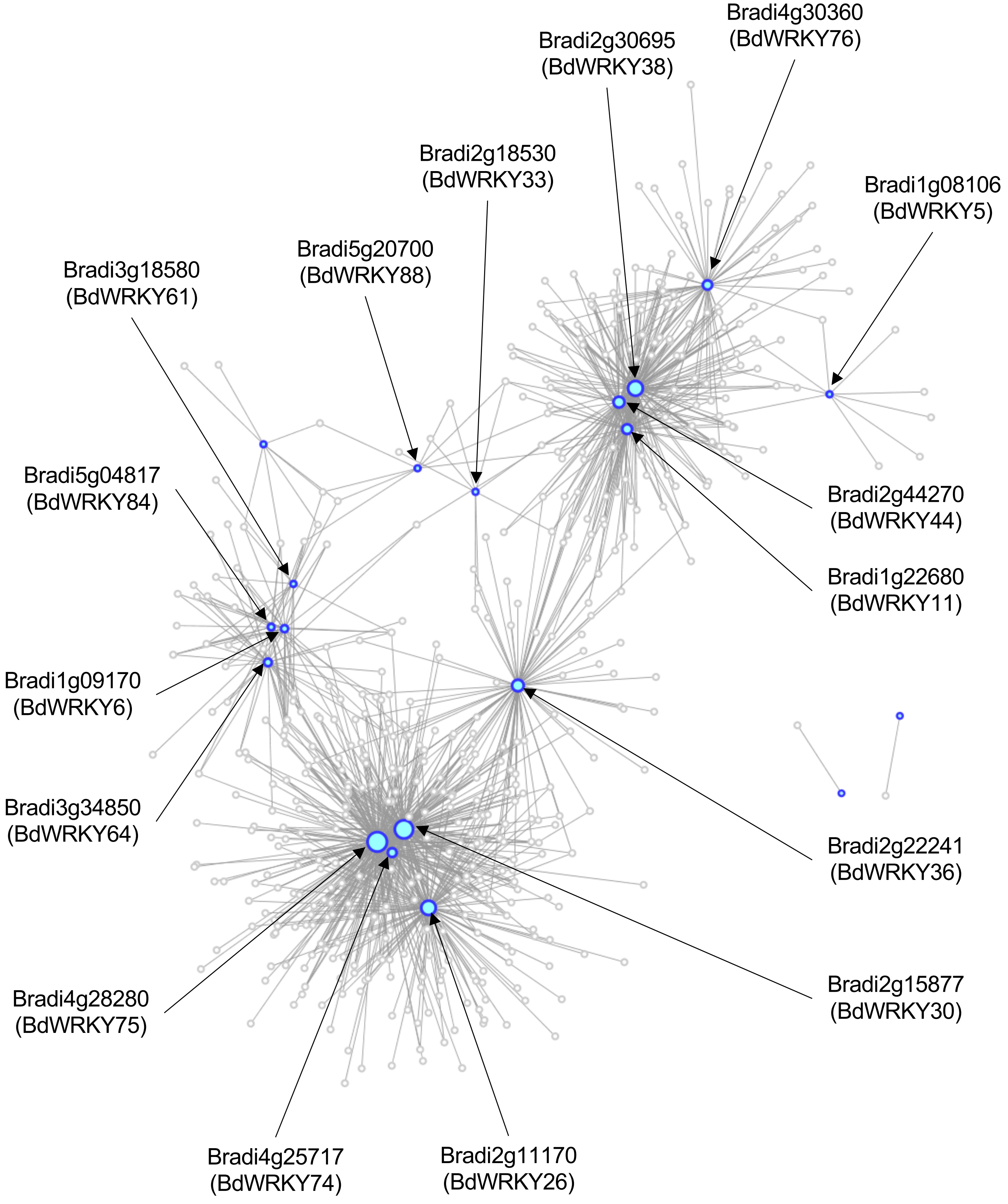

Bradi4g30360
(BdWRKY76)
Bradi2g30695
(BdWRKY38)
Bradi2g18530
(BdWRKY33)
Bradi1g08106
(BdWRKY5)
Bradi5g20700
(BdWRKY88)
Bradi3g18580
(BdWRKY61)
Bradi5g04817
(BdWRKY84)
Bradi2g44270
(BdWRKY44)
Bradi1g22680
(BdWRKY11)
Bradi1g09170
(BdWRKY6)
Bradi3g34850
(BdWRKY64)
Bradi2g22241
(BdWRKY36)
Bradi4g28280
(BdWRKY75)
Bradi2g15877
(BdWRKY30)
Bradi4g25717
(BdWRKY74)
Bradi2g11170
(BdWRKY26)
