# Supplementary material for: BdWRKY38 is required for the incompatible interaction of Brachypodium distachyon with the necrotrophic fungus Rhizoctonia solani
Source: Plant J. 2020 Sep 19;104(4):995–1008. doi: 10.1111/tpj.14976 (PMC7756360; doi:10.1111/tpj.14976)
Supplement: Supplementary file 2 — Table S1. Mapping results of time‐series RNA‐seq reads. Table S2. Hub WRKY genes in GRNs. Table S3. Mapping results of 3' mRNA‐seq reads. Table S4. Overlapped genes between potential target genes of BdWRKY38 during R. solani infection and DEGs in BdWRKY38‐ox plants. Table S5. Primers used in this study. [file TPJ-104-995-s002.docx]

# Supporting tables

## Table S1 Mapping results of time-series RNA-seq reads

| **Accession** | **Time (hpi)** | **Replicates** | **Number of total reads** | **Number of mapped reads** | **Percentage of mapped reads** | **Percentage pf properly paired reads** | **Read length (bp)** |
| --- | --- | --- | --- | --- | --- | --- | --- |
| Bd21 | 0 | 1 | 18,490,008 | 17,521,282 | 94.76 | 82.35 | 100 |
|  |  | 2 | 18,851,498 | 17,935,979 | 95.14 | 83.68 |  |
|  |  | 3 | 15,850,276 | 15,043,264 | 94.91 | 89.25 |  |
|  | 4 | 1 | 16,202,792 | 15,333,013 | 94.63 | 88.89 |  |
|  |  | 2 | 18,126,290 | 17,169,364 | 94.72 | 90.18 |  |
|  |  | 3 | 16,128,694 | 15,265,821 | 94.65 | 88.72 |  |
|  | 8 | 1 | 20,320,808 | 18,729,024 | 92.17 | 88.97 |  |
|  |  | 2 | 17,859,374 | 16,798,216 | 94.06 | 84.44 |  |
|  |  | 3 | 18,472,608 | 17,508,966 | 94.78 | 88.69 |  |
|  | 16 | 1 | 18,760,054 | 16,433,988 | 87.60 | 83.45 |  |
|  |  | 2 | 17,413,118 | 15,233,144 | 87.48 | 83.16 |  |
|  |  | 3 | 17,365,366 | 15,218,379 | 87.64 | 83.18 |  |
|  | 24 | 1 | 24,708,110 | 21,447,403 | 86.80 | 82.38 |  |
|  |  | 2 | 19,213,878 | 17,109,138 | 89.05 | 78.81 |  |
|  |  | 3 | 18,197,354 | 16,472,202 | 90.52 | 85.86 |  |
| Bd3-1 | 0 | 1 | 18,083,798 | 16,686,603 | 92.27 | 87.22 | 100 |
|  |  | 2 | 16,261,560 | 15,230,649 | 93.66 | 85.92 |  |
|  |  | 3 | 25,303,442 | 23,706,970 | 93.69 | 87.64 |  |
|  | 4 | 1 | 18,160,574 | 17,073,984 | 94.02 | 80.78 |  |
|  |  | 2 | 16,810,262 | 15,701,200 | 93.40 | 81.08 |  |
|  |  | 3 | 18,417,464 | 16,871,614 | 91.61 | 88.34 |  |
|  | 8 | 1 | 15,154,280 | 13,853,929 | 91.42 | 88.36 |  |
|  |  | 2 | 16,433,828 | 15,181,923 | 92.38 | 88.42 |  |
|  |  | 3 | 17,863,672 | 16,665,703 | 93.29 | 83.27 |  |
|  | 16 | 1 | 19,784,996 | 18,279,776 | 92.39 | 85.74 |  |
|  |  | 2 | 19,818,634 | 18,316,219 | 92.42 | 82.42 |  |
|  |  | 3 | 15,872,584 | 14,764,411 | 93.02 | 87.73 |  |
|  | 24 | 1 | 14,961,074 | 13,742,573 | 91.86 | 87.02 |  |
|  |  | 2 | 17,510,774 | 16,046,907 | 91.64 | 87.61 |  |
|  |  | 3 | 19,330,202 | 17,905,613 | 92.63 | 87.09 |  |
| Tek-3 | 0 | 1 | 29,473,850 | 26,820,394 | 91.00 | 86.53 | 100 |
|  |  | 2 | 25,895,284 | 23,693,735 | 91.50 | 85.37 |  |
|  |  | 3 | 21,649,368 | 19,946,606 | 92.13 | 83.46 |  |
|  | 4 | 1 | 27,173,624 | 24,916,974 | 91.70 | 81.03 |  |
|  |  | 2 | 22,629,786 | 20,879,060 | 92.26 | 78.55 |  |
|  |  | 3 | 23,494,028 | 21,480,415 | 91.43 | 84.55 |  |
|  | 8 | 1 | 28,554,912 | 25,863,820 | 90.58 | 85.81 |  |
|  |  | 2 | 27,368,506 | 25,110,913 | 91.75 | 81.17 |  |
|  |  | 3 | 29,619,856 | 26,695,593 | 90.13 | 84.93 |  |
|  | 16 | 1 | 25,564,934 | 23,039,688 | 90.12 | 83.35 |  |
|  |  | 2 | 25,534,656 | 22,977,478 | 89.99 | 78.90 |  |
|  |  | 3 | 24,806,704 | 22,508,628 | 90.74 | 78.52 |  |
|  | 24 | 1 | 20,067,388 | 18,037,906 | 89.89 | 80.59 |  |
|  |  | 2 | 23,226,000 | 20,985,079 | 90.35 | 77.00 |  |
|  |  | 3 | 24,315,128 | 21,861,734 | 89.91 | 75.86 |  |

## Table S2 Hub WRKY genes in gene regulatory networks

| **Bd21** |  |  |  |  |  |  |  |  |  |
| --- | --- | --- | --- | --- | --- | --- | --- | --- | --- |
| **Gene id** | **Name** | **Betweenness centrality** | **Node degree** | **Expression levels (Z-score) and assigned DYG clusters** | | | | | |
|  |  |  |  | **0h** | **4h** | **8h** | **16h** | **24h** | **Cluster** |
| Bradi1g22680 | BdWRKY11 | 0.50071 | 214 | -0.762 | -0.185 | 1.610 | 0.204 | -0.866 | 3 |
| Bradi1g08106 | BdWRKY5 | 0.43947 | 203 | -0.779 | -0.675 | 0.086 | 1.683 | -0.315 | 4 |
| Bradi2g49906 | BdWRKY50 | 0.19917 | 188 | -0.949 | -0.945 | 0.328 | 1.443 | 0.123 | 4 |
| Bradi4g25717 | BdWRKY74 | 0.12234 | 92 | -0.808 | -0.828 | -0.316 | 1.549 | 0.403 | 4 |
| Bradi2g44270 | BdWRKY44 | 0.11711 | 87 | -0.830 | -1.122 | -0.071 | 1.018 | 1.005 | 5 |
| Bradi4g30360 | BdWRKY76 | 0.13795 | 62 | -0.307 | -0.706 | 0.154 | -0.798 | 1.657 | 6 |
| Bradi1g09170 | BdWRKY6 | 0.04073 | 48 | -1.199 | -0.659 | -0.093 | 1.303 | 0.649 | 5 |
| Bradi2g00280 | BdWRKY21 | 0.05046 | 41 | -0.882 | -0.818 | -0.459 | 1.168 | 0.990 | 5 |
| Bradi2g11170 | BdWRKY26 | 0.02661 | 41 | -1.107 | -0.821 | -0.114 | 1.048 | 0.995 | 5 |
| Bradi2g42023 | BdWRKY41 | 0.03212 | 12 | -0.735 | -0.602 | -0.629 | 0.363 | 1.603 | 6 |
| **Bd3-1** |  |  |  |  |  |  |  |  |  |
| **Gene id** | **Name** | **Betweenness centrality** | **Node degree** | **Expression levels (Z-score) and assigned DYG clusters** | | | | | |
|  |  |  |  | **0h** | **4h** | **8h** | **16h** | **24h** | **Cluster** |
| Bradi2g22241 | BdWRKY36 | 0.63704 | 443 | -0.152 | -0.725 | -0.638 | 1.731 | -0.216 | 4 |
| Bradi1g08106 | BdWRKY5 | 0.39592 | 381 | -1.283 | -0.704 | 0.061 | 0.983 | 0.942 | 5 |
| Bradi2g30695 | BdWRKY38 | 0.26483 | 66 | -0.091 | 0.190 | 1.588 | -0.778 | -0.910 | 3 |
| Bradi4g30360 | BdWRKY76 | 0.14410 | 59 | -0.498 | -0.190 | 1.154 | 0.834 | -1.300 | 3 |
| Bradi1g22680 | BdWRKY11 | 0.08365 | 22 | -0.651 | 0.029 | 1.705 | -0.336 | -0.746 | 3 |
| Bradi2g42023 | BdWRKY41 | 0.03455 | 13 | -1.160 | -0.374 | -0.427 | 0.530 | 1.432 | 6 |
| Bradi2g08620 | BdWRKY25 | 0.01163 | 12 | -1.322 | -0.639 | 0.992 | 0.927 | 0.043 | 3 |
| Bradi2g44270 | BdWRKY44 | 0.01159 | 5 | -0.801 | -0.286 | 1.684 | -0.660 | 0.063 | 3 |
| **Tek-3** |  |  |  |  |  |  |  |  |  |
| **Gene id** | **Name** | **Betweenness centrality** | **Node degree** | **Expression levels (Z-score) and assigned DYG clusters** | | | | | |
|  |  |  |  | **0h** | **4h** | **8h** | **16h** | **24h** | **Cluster** |
| Bradi4g28280 | BdWRKY75 | 0.27224 | 178 | -1.016 | -0.651 | -0.354 | 0.577 | 1.444 | 6 |
| Bradi2g15877 | BdWRKY30 | 0.48413 | 163 | -1.278 | -0.072 | -0.609 | 0.788 | 1.171 | 5 |
| Bradi2g30695 | BdWRKY38 | 0.38407 | 120 | -0.156 | 0.743 | 1.067 | -0.149 | -1.504 | 3 |
| Bradi2g11170 | BdWRKY26 | 0.17413 | 115 | -1.291 | -0.715 | 0.175 | 1.154 | 0.677 | 5 |
| Bradi2g22241 | BdWRKY36 | 0.21374 | 81 | -0.473 | -0.422 | -0.581 | 1.780 | -0.304 | 4 |
| Bradi2g44270 | BdWRKY44 | 0.03581 | 72 | -0.985 | 0.473 | 1.489 | -0.251 | -0.726 | 3 |
| Bradi1g22680 | BdWRKY11 | 0.05737 | 58 | -0.676 | 0.081 | 1.505 | 0.200 | -1.110 | 3 |
| Bradi4g30360 | BdWRKY76 | 0.08025 | 52 | -0.321 | 0.889 | -0.078 | 0.976 | -1.466 | 3 |
| Bradi4g25717 | BdWRKY74 | 0.03553 | 47 | -1.408 | -0.532 | 0.326 | 1.210 | 0.405 | 5 |
| Bradi3g34850 | BdWRKY64 | 0.05544 | 32 | -1.402 | -0.228 | 0.252 | -0.007 | 1.385 | 6 |
| Bradi1g09170 | BdWRKY6 | 0.04087 | 26 | -1.370 | -0.589 | 0.462 | 0.278 | 1.219 | 6 |
| Bradi5g04817 | BdWRKY84 | 0.01904 | 20 | -1.467 | -0.412 | 0.492 | 0.205 | 1.181 | 6 |
| Bradi3g18580 | BdWRKY61 | 0.01253 | 11 | -1.222 | -0.770 | 0.818 | 0.059 | 1.115 | 6 |
| Bradi1g08106 | BdWRKY5 | 0.01896 | 9 | -1.327 | -0.190 | 0.521 | 1.345 | -0.349 | 4 |
| Bradi5g20700 | BdWRKY88 | 0.01312 | 8 | -1.392 | -0.312 | 1.288 | -0.123 | 0.538 | 6 |
| Bradi2g18530 | BdWRKY33 | 0.01134 | 8 | -1.103 | -0.529 | 1.521 | 0.358 | -0.247 | 3 |

## Table S3 Mapping results of 3' mRNA-seq reads

| **Line** | **Replicates** | **Number of total reads** | **Number of mapped reads** | **Percentage of mapped reads** |
| --- | --- | --- | --- | --- |
| Bd21 (WT) | 1 | 18,693,392 | 15,495,935 | 82.90 |
|  | 2 | 16,839,891 | 14,200,833 | 84.33 |
|  | 3 | 16,584,996 | 13,989,579 | 84.35 |
| BdWRKY38-ox | 1 | 18,279,846 | 15,668,144 | 85.71 |
|  | 2 | 13,351,524 | 11,170,140 | 83.66 |
|  | 3 | 12,086,634 | 10,430,760 | 86.30 |

## Table S4 Overlapped genes between potential target genes of BdWRKY38 during *R. solani* infection and differentially expressed genes in BdWRKY38-ox plants

| **Gene Id** | **Name** | **RPM** | | **LogFC** | **FDR value** | **Homologs in rice genome** |
| --- | --- | --- | --- | --- | --- | --- |
|  |  | **Bd21 (WT)** | **BdWRKY38-ox** |  |  |  |
| Bradi4g30360 | BdWRKY76 | 0.62 | 22.03 | 4.91 | 7.08E-05 | LOC_Os09g25060.1 \| WRKY76, expressed |
| Bradi2g44270 | BdWRKY44 | 1.20 | 7.36 | 2.36 | 1.82E-03 | LOC_Os05g25770.1 \| WRKY45, expressed |
| Bradi1g42880 | N/A | 2.72 | 16.14 | 2.32 | 1.53E-07 | LOC_Os05g19150.1 \| hydrolase, alpha/beta fold family domain containing protein, expressed |
| Bradi2g45143 | N/A | 10.60 | 55.55 | 2.09 | 9.65E-04 | LOC_Os01g45914.1 \| expressed protein |
| Bradi5g13660 | N/A | 28.72 | 122.23 | 1.84 | 9.53E-15 | LOC_Os04g40600.1 \| peptide methionine sulfoxide reductase, putative, expressed |
| Bradi1g14700 | N/A | 3.55 | 10.12 | 1.23 | 1.37E-03 | LOC_Os03g41510.1 \| oxidoreductase, aldo/keto reductase family protein, putative, expressed |
| Bradi1g75950 | N/A | 22.07 | 59.32 | 1.16 | 1.80E-07 | LOC_Os04g01310.1 \| serine/threonine-protein kinase receptor precursor, putative, expressed |
| Bradi1g20930 | N/A | 27.40 | 70.24 | 1.10 | 2.00E-04 | LOC_Os07g42570.1 \| dirigent, putative, expressed |
| Bradi1g09410 | N/A | 27.07 | 66.62 | 1.05 | 3.80E-02 | LOC_Os03g52680.1 \| expressed protein |
| Bradi2g46502 | N/A | 47.57 | 117.41 | 1.05 | 7.96E-04 | LOC_Os01g48640.1 \| expressed protein |
| Bradi1g57750 | N/A | 22.14 | 51.71 | 0.98 | 6.08E-04 | LOC_Os09g09500.1 \| lectin-like receptor kinase, putative, expressed |
| Bradi1g32920 | N/A | 8.03 | 16.38 | 0.77 | 1.84E-02 | LOC_Os02g19820.1 \| nodulin MtN3 family protein, putative, expressed |
| Bradi3g36500 | N/A | 26.50 | 51.96 | 0.71 | 8.36E-04 | LOC_Os08g33420.1 \| agenet domain containing protein, expressed |
| Bradi4g24650 | N/A | 155.80 | 304.46 | 0.71 | 3.55E-04 | LOC_Os11g06720.1 \| abscisic stress-ripening, putative, expressed |
| Bradi2g52790 | N/A | 23.43 | 41.17 | 0.55 | 1.86E-02 | LOC_Os01g59150.1 \| tubulin/FtsZ domain containing protein, putative, expressed |
| Bradi4g08097 | N/A | 62.73 | 52.76 | -0.52 | 7.99E-03 | LOC_Os12g22030.1 \| serine hydroxymethyltransferase, mitochondrial precursor, putative, expressed |
| Bradi2g44760 | N/A | 25.69 | 20.55 | -0.59 | 3.20E-02 | LOC_Os01g45070.1 \| peptide deformylase, putative, expressed |
| Bradi5g17170 | N/A | 28.65 | 21.80 | -0.65 | 8.12E-03 | LOC_Os04g45810.1 \| homeobox associated leucine zipper, putative, expressed |
| Bradi3g13160 | N/A | 520.09 | 385.68 | -0.70 | 1.47E-05 | LOC_Os08g01380.1 \| 2Fe-2S iron-sulfur cluster binding domain containing protein, expressed |
| Bradi3g48490 | N/A | 25.91 | 18.81 | -0.73 | 7.91E-03 | LOC_Os02g39790.1 \| CPuORF9 - conserved peptide uORF-containing transcript, expressed |
| Bradi3g11060 | N/A | 37.63 | 22.88 | -0.97 | 9.60E-05 | LOC_Os10g35840.1 \| shikimate/quinate 5-dehydrogenase, putative, expressed |
| Bradi2g56750 | N/A | 10.79 | 6.12 | -1.08 | 1.18E-03 | LOC_Os01g65650.1 \| receptor-like protein kinase HAIKU2 precursor, putative, expressed |
| Bradi2g07160 | N/A | 31.83 | 17.52 | -1.13 | 3.21E-07 | LOC_Os01g12080.1 \| plant-specific domain TIGR01589 family protein, putative, expressed |
| Bradi2g60705 | N/A | 86.89 | 27.42 | -1.98 | 7.28E-08 | LOC_Os05g31470.1 \| expressed protein |

## Table S5 Primers used in this study.

| **Name** | **Sequence (5' to 3')** | **Target** |
| --- | --- | --- |
| **1) Fungal biomass quantification** | |  |
| Rs-1F | GCCTTTTCTACCTTAATTTGGCAG | *R. solani* AG-1, 1A rDNA (Sayler & Yang, 2007) |
| Rs-2R | GTGTGTAAATTAAGTAGACAGCAAATG |  |
| BdFIM-F | CCTCACACGGATTTCGAGAGA | *Bradi2g13800, BdFIM* (Zhu *et al*. 2014) |
| BdFIM-R | GGACAACCCATTTCTGCGA |  |
| **2) Vector construction for *WRKY*-knockdown plants** | |  |
| 2g30695-FW2 | caccACTCCTACCACGACGACAA | The 359 bp fragment located to the 3’ region of *Bradi2g30695, BdWRKY38* |
| 2g30695-RV1 | TAGTGGTTGCGGCTCGAGAA |  |
| 2g44270 -FW2 | caccACCACGAGCGTGACCACAAA | The 447 bp fragment located to the 3’ region of *Bradi2g44270, BdWRKY44* |
| 2g44270-RV1 | AGCTAGCAATAGCACACCTGC |  |
| **3) Vector construction for *NahG-*overexpressing plants** | |  |
| NahG-FW1 | ggatccATGCAGAACTCGACCAGTGC | The full-length synthetic sequence of *NahG* |
| NahG-RV1 | actagtTCAGGCAGCCTGGGCGCGTA |  |
| **4) Vector construction for *BdWRKY38*-overexpressing plants** | |  |
| IF-30695-F | ACAGGGATCCGTCGAATGGCGTGGCCGCCGCCG | The full-length CDS sequence of *Bradi2g30695*, *BdWRKY38* |
| IF-30695-R | TTGGCTGCAGGTCGATCAACCATCGAGATCGAAC |  |
| **5) Gene expression analysis** | |  |
| W45L1-F | GGACACCTTCAGGGTGACAT | *Bradi2g30695,* *BdWRKY38* |
| W45L1-R | TTGTCGTCGTGGTAGGAGTG |  |
| W45L2-F | GATCGGAGGTGCAGAGAGAG | *Bradi2g44270,* *BdWRKY44* |
| W45L2-R | GTGTGCACCGGAAGTAGGAT |  |
| NahG_Check-F | CCTCTCTGTGGGCTCTTCAC | The ectopically expressed *NahG* gene |
| NahG_Check-R | AGGAGTCTAGCGAGCAACCA |  |
| BdUbi4-3F | GCTGTTGGAACTGCTGCTATACCT | *Bradi3g04730, BdUbi4* (Chambers *et al.* 2012) |
| BdUbi4-3R | TTGCACCAAACCAACACACACCAG |  |
